# QTL mapping and characterization of black spot disease resistance using two multi-parental diploid rose populations

**DOI:** 10.1093/hr/uhac183

**Published:** 2022-08-25

**Authors:** Zena J Rawandoozi, Ellen L Young, Muqing Yan, Seza Noyan, Qiuyi Fu, Tessa Hochhaus, Maad Y Rawandoozi, Patricia E Klein, David H Byrne, Oscar Riera-Lizarazu

**Affiliations:** Department of Horticultural Sciences, Texas A&M University, College Station, TX 77843, USA; Department of Horticultural Sciences, Texas A&M University, College Station, TX 77843, USA; Department of Horticultural Sciences, Texas A&M University, College Station, TX 77843, USA; Department of Horticultural Sciences, Texas A&M University, College Station, TX 77843, USA; Department of Horticultural Sciences, Texas A&M University, College Station, TX 77843, USA; Department of Horticultural Sciences, Texas A&M University, College Station, TX 77843, USA; Norman Borlaug Institute for International Agriculture and Development, Texas A&M AgriLife Research, Texas A&M System, College Station, TX, 77843 USA; Department of Horticultural Sciences, Texas A&M University, College Station, TX 77843, USA; Department of Horticultural Sciences, Texas A&M University, College Station, TX 77843, USA; Department of Horticultural Sciences, Texas A&M University, College Station, TX 77843, USA

## Abstract

Black spot disease (BSD) (*Diplocarpon rosae*) is the most common and damaging fungal disease in garden roses (*Rosa sp*.). Although qualitative resistance to BSD has been extensively investigated, the research on quantitative resistance lags behind. The goal of this research was to study the genetic basis of BSD resistance in two multi-parental populations (TX2WOB and TX2WSE) through a pedigree-based analysis approach (PBA). Both populations were genotyped and evaluated for BSD incidence over five years in three locations in Texas. A total of 28 QTLs, distributed over all linkage groups (LGs), were detected across both populations. Consistent minor effect QTLs included two on LG1 and LG3 (TX2WOB and TX2WSE), two on LG4 and LG5 (TX2WSE), and one QTL on LG7 (TX2WOB). In addition, one major QTL detected in both populations was consistently mapped on LG3. This QTL was localized to an interval ranging from 18.9 to 27.8 Mbp on the *Rosa chinensis* genome and explained 20 and 33% of the phenotypic variation. Furthermore, haplotype analysis showed that this QTL had three distinct functional alleles. The parent PP-J14–3 was the common source of the LG3 BSD resistance in both populations. Taken together, this research presents the characterization of new SNP-tagged genetic determinants of BSD resistance, the discovery of marker-trait associations to enable parental choice based on their BSD resistance QTL haplotypes, and substrates for the development of trait-predictive DNA tests for routine use in marker-assisted breeding for BSD resistance.

## Introduction

Roses (*Rosa* sp.) are woody perennial plants belonging to the Rosaceae family, which includes many economically important fruit and ornamental crops. The production of cultivated roses is valued at $28 billion globally [1]. In the USA, garden rose production has decreased from $203 million in 2014 to $168 million in 2019 [2, 3]. This decrease is, in part, due to the susceptibility of roses to a broad range of diseases that can cause plant death or negatively impact the ornamental and market value as the infected plants become unattractive [4]. A survey of the rose industry and community showed that disease resistance was more important than ornamental traits in new cultivars [5].

Black spot disease (BSD) is the most widespread rose foliar disease in the world. The causal agent of BSD in roses is the hemibiotrophic ascomycete fungus *Diplocarpon rosae* Wolf. The development of this disease is favored by warm, humid environments and its spread by rain-induced water splash. BSD infected plants show dark spots with feathery margins, often followed by chlorosis, plant weakening, and, on very susceptible genotypes, complete defoliation, plant dieback, and possibly death [1]. Most modern rose cultivars are susceptible to this disease.

Fungicides are frequently used to control this disease [6, 7]. Nevertheless, excessive use of fungicides leads to the development of pesticide-resistant pathogens and restrictions on agrochemical use due to environmental and public health concerns [1]. This, combined with the consumers’ demand for rose plants with natural resistance, has pushed most garden rose breeding programs to prioritize the development of disease-resistant cultivars [8]. At present, the phenotypic assessment of disease resistance is slow as it relies on multi-year and multi-location field trials to properly expose the roses to a broad range of pathogenic races [1]. Thus, DNA-informed breeding needs to be incorporated into traditional breeding operations to accelerate the introgression of disease resistance genes into commercial germplasm. However, incorporating molecular tools in rose breeding lags behind many crops because rose is a complex, highly heterozygous crop with multiple ploidy levels [9, 10].

Qualitative or complete (conferred by major dominant genes) and quantitative or partial (conferred by multiple genes) resistance to BSD has been reported in roses. Four *Rdr* (resistance to *D. rosae*) dominant genes (*Rdr1*, *Rdr2*, *Rdr3*, and *Rdr4*) have been described for BSD in roses [11, 12]. These are located on chromosomes 1 (*Rdr1* and *Rdr2*) [12–15], 6 (*Rdr3*) [16], and 5 (*Rdr4*) [14]. Partial or horizontal resistance to BSD, which is controlled by multiple genes and/or quantitative trait loci (QTL) [17, 18], can be effective and durable over a broad spectrum of pathogenic races [11, 18]. BSD resistance was reported to have low narrow-sense heritability (h^2^) and moderate to high broad-sense heritability (H^2^) [9, 18, 19]. A pedigree-based QTL mapping approach using 15 interrelated diploid families with *Rosa wichurana* Crép. background detected a major QTL associated with BSD on LG3 which explained ~13% of total phenotypic variance [19]. Additional QTL studies also using populations with *R. wichurana* in their parentage have identified QTLs on chromosomes 3 and 5 [20–22].

The pedigree-based analysis (PBA) approach developed for highly heterozygous crops has key advantages over the use of bi-parental populations for QTL mapping. For one, PBA utilizes multiple pedigree-connected families that increase the ability to detect QTLs and alleles with major and minor effects across various genetic backgrounds. The analysis also yields SNPs and haplotypes associated with the QTL and the sources of a given QTL allele. This information would enable the selection of parents and seedlings with favorable QTL alleles.

Thus, this study is distinguished from previous work by exploiting the joint analysis of multi-parental populations through FlexQTL [23] to detect new and/or previously reported QTLs for BSD, and by the use of recent tools like polymapR [24] to construct high-density linkage maps from highly heterozygous parents. The findings of this research will help rose breeders by identifying SNP markers for QTL selection and tracking as well as QTL characterized germplasm with resistance alleles for subsequent breeding.

This study aims to 1.) construct two consensus maps for three and five diploid rose populations; 2.) identify QTLs associated with BSD resistance using two different multi-parental populations; 3.) determine the QTL genotype of parents; and 4.) identify predictive SNP marker(s) associated with QTL alleles that either decrease or increase resistance.

## Results

### Phenotypic data analysis

The TX2WOB multi-parental population (11 populations with nine parents) was evaluated for BSD incidence in two locations in Texas, College Station (CS) in 2016 and Somerville (SV) in 2019 and 2021 (Table S3). In evaluations involving the TX2WOB population, the mean BSD score (0–9 rating scale) in CS 2016 was the lowest in Sep. (2.45) and the highest in June, Oct., and Nov. (3.60 and 3.50) (Table S3). BSD severity was skewed towards zero in Sep. and towards higher ratings in both Oct. and Nov. It was more normally distributed in June (Fig. S3A). BSD severity was low (1.5) in Nov. in SV 2019 and normally distributed (Fig. S3B). The lowest mean disease severity in SV 2021 was observed in June (2.0), and the highest in Nov. (3.1) (Table S3). Similarly, most plants in this year had low BSD and the data was skewed towards lower BSD ratings (Fig. S3C).

The TX2WSE multi-parental population (six populations from crosses of nine parents) was evaluated for BSD incidence in two locations in Texas, Overton (OV) 2019 and Somerville (SV) in 2018 and 2020 (Table S5). Regarding evaluations of the TX2WSE population in the SV 2018 and SV 2020 environments, the BSD incidence means of Nov. (2.4) and July (4.4) were the highest (Table S5), respectively. BSD data was skewed towards lower BSD ratings in SV 2018 (Fig. S5A). However, data appeared normally distributed in SV 2020, except for May (Fig. S5B).

In OV 2019, BSD followed a different pattern as the severity rates were highest in June (1.9) and lowest in Sep. (1.1) (Table S5). No dataset was normally distributed, and all were skewed towards lower BSD ratings (Fig. S5C). Similarly, in this dataset, the BSD incidence in SV 2018 and OV 2019 was noticeably low.

### Genotype by environment interactions

Understanding the genotype × environment interaction (G × E) is important when studying complex traits. In this analysis, the SV 2019 environment was excluded from datasets from the TX2WOB population because of insufficient disease pressure due to the lack of either the initial inoculum and/or the appropriate environmental conditions to encourage disease development.

BSD resistance had low to moderate broad-sense heritability (H^2^) (from 0.39 to 0.57) and high G × E variance ratio $\Big({\sigma}_{g\times e}^2/{\sigma}_g^2\Big)$ (10.76 to 6.10) (Table S6) in the TX2WOB and TX2WSE populations, respectively. The high G × E may have resulted from low BSD levels in SV 2021, SV 2018, and OV 2019. These findings imply that BSD incidence is highly influenced by the environment, which is supported by the GGE biplot that showed high PC2 scores ranging from 9.03 to 15.19% (Fig. S6A and B) and very low negative (r = −0.13) to moderate positive correlations (r = 0.39–0.55) among the BSD incidence in the different environments (Table S7).

GGE biplots with data from the TX2WOB population showed that CS 2016 had a longer environmental vector than SV 2021 (Fig. S6A), implying that CS 2016 had greater discrimination among genotypes for BSD incidence. This could have resulted from the difference in the progeny numbers (297 vs. 721) tested (Table S1), plant age (Fig. S4), rainfall from May through Nov. (~549 vs.736 mm), or humidity (Table S4) in these years. However, in data from the TX2WSE population, OV 2019 showed the shorter vector (Fig. S6B), which suggests this environment had less discrimination among genotypes. In contrast, SV 2018 and SV 2020 equally discriminated genotypes for BSD incidence, indicated by the similar length of the environmental vectors. SV 2018 was far from the other environments, suggesting that this environment discriminates the genotypes differently, which is also confirmed by the lower correlation coefficient of SV 2018 with other environments (Table S7).

### Consensus maps

For the TX2WOB multi-parental population, 415 individuals from five populations were used to construct linkage maps with 14 706 to 21 055 markers per population. The final integrated consensus map (TX2WOB ICM) contained 4467 markers with 3247 SNP markers shared between at least two populations (1896 markers in unique positions) and with a density of 6.9 markers/cM (2.9 in unique positions), distributed over 653.1 cM (Table S8, Fig. S7, and S8). LG2 had the most markers, whereas LG7 had the least. LG5 was the longest, and LG3 was the shortest. The TX2WOB ICM showed very high collinearity with the rose genome [25] with a Spearman’s correlation coefficient of 0.99 (data not shown).

For the TX2WSE multi-parental population, 314 individuals from three populations were used to construct linkage maps with 5239 to 9408 markers each. An integrated consensus map (TX2WSE ICM) was developed with 2677 markers in which 1378 were common between at least two populations. This TX2WSE ICM had a length of 758.2 cM with a density of 3.5 markers/cM (1.5 unique positions/cM). LG2 had the most markers and was the longest, while LG4 had the least number of markers and LG6 was the shortest. The maximum gap was 6.8 cM on LG7 (Table S9, Fig. S9, and S10). As collinearity with the rose genome was an assumption of the “SE” imputation process, collinearity was not calculated for this map. After further curation, a total of 1115 and 866 informative SNP markers were used for QTL mapping with the TX2WOB and TX2WSE multi-parental populations, respectively.

### Narrow-sense heritability (h^2^)

Narrow-sense heritability was estimated from FlexQTL outputs for BSD resistance. The h^2^ of BSD resistance ranged from low to moderate among environments in data for the TX2WOB population (Table S10). The lowest h^2^ (0.17) was observed in Nov. of SV 2019, whereas the largest h^2^ (0.43) was observed in CS 2016. Similarly, the h^2^ of BSD resistance in data from the TX2WSE population ranged from low to moderate (Table S11). The lowest h^2^ (0.17) was observed in May and July in SV 2018 and SV 2020, while the largest h^2^ value (0.41) was observed in SV 2018 and June of OV 2019.

### Genome-wide QTL analysis

Using FlexQTL for QTL mapping, a total of 13 QTLs were mapped on all LGs using the TX2WOB population (721 progenies and nine parents) and ten environments across two locations and three years (Table S10, Fig. S11, and S12). Meanwhile, 15 QTLs were mapped across all LGs except LG7 using the TX2WSE population (378 progenies and nine parents) and 12 environments over two locations and three years (Table 2, Fig. S14, S15, and S16).

All mapped QTLs in this study were compared across datasets (month, year, and location), and those consistently co-localized were considered the same QTLs.

**Figure 1:**
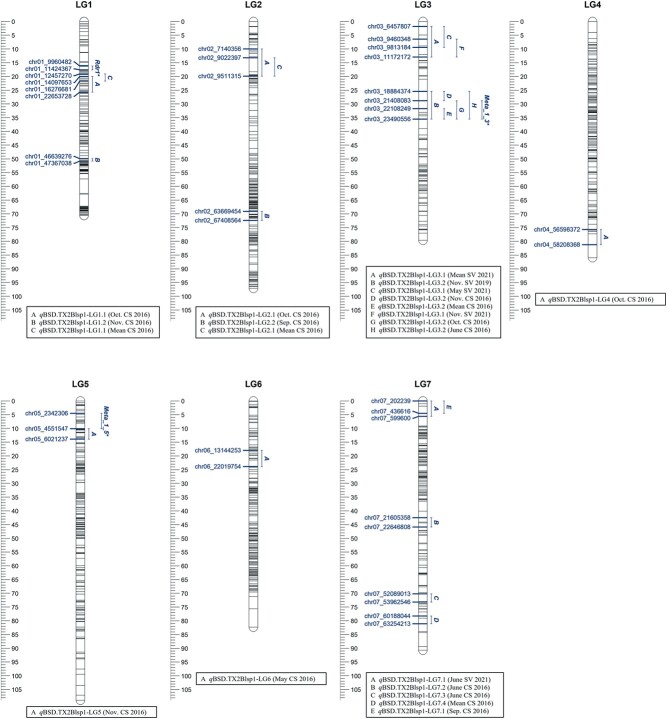
Positions of putative QTLs controlling the black spot disease (BSD) severity across 11 diploid rose populations at linkage groups (LG) of the five-population (TX2WOB) consensus map. QTL names are listed below each LG. The plot was generated using MapChart 2.32.

In the analysis of data from TX2WOB, one major QTL was discovered consistently on LG3 (*q*BSD.TX2WOB-LG3.2) over five environments in 2016 and 2019 with positive, strong, and decisive evidence and high posterior intensity (Table S10, Fig. S11, and S12). *q*BSD.TX2WOB-LG3.2 was localized to an interval between 25.4 and 35.5 cM (peaks 25, 29, 30, 32, and 35 cM), and 18.8 and 23.4 Mbp on the rose genome (Table 1 and Fig. 1). The proportion of the variance explained (PVE) by this QTL was between 12–20% except for June (Table 1). In this study, *q*BSD.TX2WOB-LG3.2 passed our inclusion criteria for further analysis. In contrast, *q*BSD.TX2WOB-LG3.1, a QTL located upstream of *q*BSD.TX2WOB-LG3.2, was environment-specific and detected only in three environments in SV 2021. *q*BSD.TX2WOB-LG3.2 had peaks at 2, 7, and 10 cM (Table 1, Fig. 1, S11, and S12). The interval of this QTL was between 1.8 and 12.9 cM (6.4 to 11.17 Mbp), with PVE ranging from 13 to 21%. (Table 1). Furthermore, *q*BSD.TX2WOB-LG7.1, at the proximal end of LG7, had an interval of 0.0–5.6 cM (0.22–0.59 Mbp) and a PVE of 15% (Table 1 and Fig. 1), was common between 2016 and 2021 environments. Two additional QTLs were only mapped in data from 2016. These QTLs were *q*BSD.TX2WOB-LG1.1 with an interval between 19.1–25.7 cM (12.4–22.6 Mbp) and a PVE of 7–9% and *q*BSD.TX2WOB-LG2.1 with an interval of 10.0–19.9 cM (7.1–9.5 Mbp) and a PVE of 8–9%. The remaining mapped QTLs were found only in data from one month.

**Table 1:** QTL name, linkage group (LG), interval, mode peak (Mode), posterior intensity (QTL intensity), phenotypic variance explained (PVE), and Bayes factor (BF) for black spot disease (BSD) evaluated in Texas on 11 rose diploid populations (TX2WOB) across multiple months and overall mean in 2016 in College Station (CS) and on a ten-population subset in 2019 and 2021 in Somerville (SV)

QTL name	Month	Year	LG	Mode	Interval		QTL	PVE	BF^a^
					(cM)	(cM)	(Mbp)	intensity	(%)
*q*BSD.TX2WOB-LG1.1	Oct.	2016	1	24	20.0	14.10	0.32	7	2.7
					25.7	22.65			
	Mean	2016	1	21	19.1	12.46	0.58	9	3.4
					21.7	16.28			
*q*BSD.TX2WOB-LG1.2	Nov.	2016	1	50	49.9	46.64	0.72	11	7.6
					50.8	47.18			
*q*BSD.TX2WOB-LG2.1	Oct.	2016	2	14	10.0	7.14	0.40	9	2.2
					19.9	9.51			
	Mean	2016	2	14	13.2	9.02	0.84	8	7.0
					19.9	9.51			
*q*BSD.TX2WOB-LG2.2	Sep.	2016	2	72	69.1	63.67	0.94	20	6.2
					72.4	67.41			
*q*BSD.TX2WOB-LG3.1	May	2021	3	2	1.8	6.45	0.88	13	6.0
					9.4	9.81			
	Nov.	2021	3	10	6.4	9.46	0.76	17	4.3
					12.9	11.17			
	Mean	2021	3	7	1.8	6.45	0.87	21	5.3
					12.9	11.17			
*q*BSD.TX2WOB-LG3.2	June	2016	3	35	31.7	22.10	0.67	5	3.2
					35.5	23.49			
	Oct.	2016	3	32	25.4	18.88	1.00	15	12.4
					35.5	23.49			
	Nov.	2016	3	25	25.4	18.88	1.07	12	13.5
					28.8	21.40			
	Mean	2016	3	30	28.8	21.40	1.05	12	29.1
					35.5	23.49			
	Nov.	2019	3	29	25.4	18.88	1.18	20	10.5
					35.5	23.49			
*q*BSD.TX2WOB-LG4	Oct.	2016	4	75	75.7	56.59	0.58	15	4.2
					81.2	58.20			
*q*BSD.TX2WOB-LG5	Nov.	2016	5	11	10.1	4.55	0.89	5	4.6
					13.9	6.02			
*q*BSD.TX2WOB-LG6	May	2021	6	22	18.0	13.14	0.88	8	4.8
					23.9	22.01			
*q*BSD.TX2WOB-LG7.1	Sep.	2016	7	1	0.0	0.20	0.95	15	7.5
					4.5	0.44			
	June	2021	7	1	0.0	0.20	0.73	15	5.6
					5.6	0.59			
*q*BSD.TX2WOB-LG7.2	June	2016	7	45	42.5	21.61	0.72	15	4.1
					45.9	22.65			
*q*BSD.TX2WOB-LG7.3	June	2016	7	72	70.2	52.09	0.53	20	3.8
					73.2	53.96			
*q*BSD.TX2WOB-LG7.4	Mean	2016	7	79	78.3	60.19	0.29	8	2.1
					81.1	63.25			

a Bayes Factor (*2lnBF)*, a measure quantifies the support from the data for the number of QTL(s) in the model (QTL evidence), after pair-wise model comparison (e.g. 1/0, 2/1, and 3/2) such as “one-QTL model” vs. ‘zero-QTL model, etc. BF ≥ 2, 5, 10 indicating positive, strong, or decisive evidence for the presence of a QTL, respectively.

The QTL genotypes at the peak of *q*BSD.TX2WOB-LG3.2 had mean BSD severity rating of 3.2, 2.6, and 1.92 for offspring with the *QQ*, *Qq*, and *qq* QTL genotypes, respectively (Fig. S13A). The unfavorable allele (*Q*), associated with increasing BSD incidence, was more prevalent in this dataset than the favorable allele (*q*).

**Figure 2:**
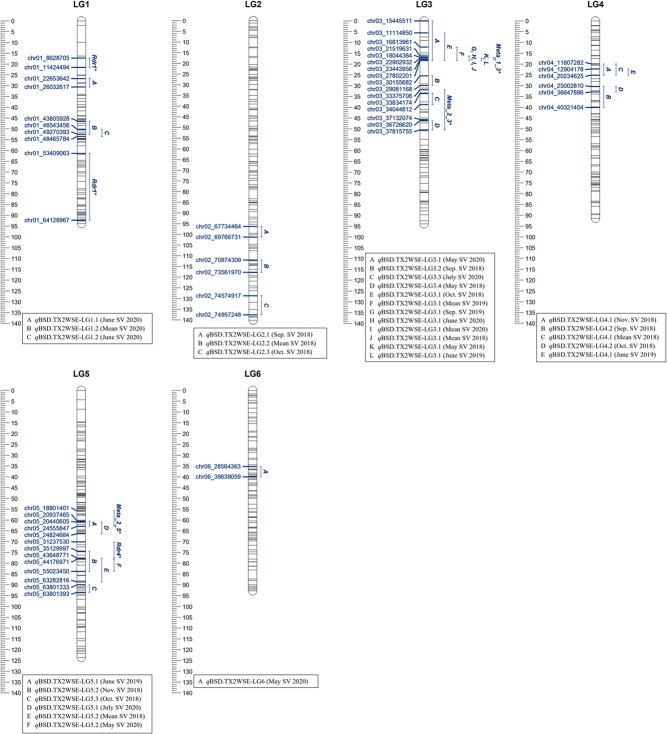
Positions of putative QTLs controlling the black spot disease (BSD) severity across six diploid rose populations at linkage groups (LG) of the three-population (TX2WSE) consensus map. QTL names are listed below each LG. The plot was generated using MapChart 2.32.

Similarly, in data from TX2WSE, a single major QTL mapped on LG3 (*q*BSD.TX2WSE-LG3.1) over nine environments from SV 2018, SV 2020, and OV 2019 (Table 2, Fig. S14, S15, and S16). The *q*BSD.TX2WSE-LG3.1 interval spanned 16.31 to 18.39 cM, or 21.51–27.80 Mbp on the rose genome in data from six environments, however, the intervals were wider in May 2020, mean 2019, and Oct. 2018 (Table 2 and Fig. 2). Peaks of this QTL co-localized at 18 cM across seven environments, while analysis of data from two environments yielded peaks at either 10 or 16 cM. Therefore, in this dataset, *q*BSD.TX2WSE-LG3.1 was considered for downstream analysis as it was stable and consistently mapped over multiple environments with strong evidence and intensity and a PVE between 15–33% in most cases (Table 2). Three additional QTLs on LG3 were discovered downstream of *q*BSD.TX2WSE-LG3.1.

**Table 2:** QTL name, linkage group (LG), interval, mode peak (Mode), posterior intensity (QTL intensity), phenotypic variance explained (PVE), and Bayes factor (BF) for black spot disease (BSD) evaluated in Texas on six diploid rose populations (TX2WSE) across multiple months and overall mean in 2018 and 2020 Somerville (SV) and 2019 in Overton (OV)

QTL name	Month	Year	LG	Mode	Interval		QTL	PVE	BF^a^
					(cM)	(cM)		(%)	
*q*BSD.TX2WSE-LG1.1	June	2020	1	30	28.73	22.65	1.10	11	6.3
					32.63	26.03			
*q*BSD.TX2WSE-LG1.2	June	2020	1	54	52.16	46.54	0.50	8	2.0
					55.52	48.46			
	Mean	2020	1	54	48.36	43.80	0.85	6	2.1
					54.51	49.27			
*q*BSD.TX2WSE-LG2.1	Sep.	2018	2	100	97.39	67.73	0.72	7	4.7
					102.35	69.76			
*q*BSD.TX2WSE-LG2.2	Mean	2018	2	114	113.12	70.87	0.81	8	3.3
					118.89	73.56			
*q*BSD.TX2WSE-LG2.3	Oct.	2018	2	137	129.77	74.57	1.10	7	6.0
					138.68	74.95			
*q*BSD.TX2WSE-LG3.1	May	2020	3	10	0.00	15.44	1.00	9	5.3
					18.39	27.80			
	Mean	2019	3	16	12.11	16.81	1.11	33	27.8
					18.39	27.80			
	June	2020	3	18	16.31	21.51	0.40	8	3.3
					18.39	27.80			
	Sep.	2019	3	18	16.31	21.51	1.03	19	6.6
					18.39	27.80			
	May	2018	3	18	17.21	22.90	1.19	24	24.9
					18.39	27.80			
	Mean	2018	3	18	16.31	21.51	0.72	15	27.9
					18.39	27.80			
	Mean	2020	3	18	16.31	21.51	1.12	21	27.7
					18.39	27.80			
	June	2019	3	18	17.21	22.90	1.09	26	27.8
					18.39	27.80			
	Oct.	2018	3	18	5.61	11.11	0.86	6	3.8
					18.39	27.80			
*q*BSD.TX2WSE-LG3.2	Sep.	2018	3	29	25.38	30.15	0.65	19	4.5
					29.88	29.08			
*q*BSD.TX2WSE-LG3.3	July	2020	3	34	33.53	33.83	1.20	8	27.2
					38.71	34.04			
*q*BSD.TX2WSE-LG3.4	May	2018	3	50	46.19	36.72	0.55	7	24.9
					50.49	37.81			
*q*BSD.TX2WSE-LG4.1	Nov.	2018	4	25	22.14	11.80	0.91	13	25.7
					27.27	20.23			
	June	2019	4	26	24.47	12.90	0.44	9	9.3
					27.27	20.23			
	Mean	2018	4	24	22.14	11.80	0.66	7	26.1
					27.27	20.23			
*q*BSD.TX2WSE-LG4.2	Oct.	2018	4	32	32.55	25.00	0.44	6	2.3
					35.07	36.64			
	Sep.	2018	4	39	32.55	25.00	0.51	8	8.1
					42.29	40.32			
*q*BSD.TX2WSE-LG5.1	June	2019	5	62	60.51	20.93	0.89	9	8.6
					62.93	24.55			
	July	2020	5	66	61.01	20.44	1.14	6	8.6
					66.26	24.82			
*q*BSD.TX2WSE-LG5.2	Nov.	2018	5	78	74.36	35.12	0.80	11	26.2
					83.82	55.02			
	May	2020	5	81	78.11	44.17	1.03	9	8.1
					83.82	55.02			
	Mean	2018	5	78	77.68	43.64	0.88	12	27.4
					88.70	63.28			
*q*BSD.TX2WSE-LG5.3	Oct.	2018	5	91	90.03	63.80	1.00	26	27.3
					93.50	63.80			
*q*BSD.TX2WSE-LG6	May	2020	6	38	35.24	28.56	1.13	15	27.1
					39.98	39.63			

a Bayes Factor (*2lnBF)*, a measure quantifies the support from the data for the number of QTL(s) in the model (QTL evidence), after pair-wise model comparison (e.g. 1/0, 2/1, and 3/2) such as “one-QTL model” vs. ‘zero-QTL model, etc. BF ≥ 2, 5, 10 indicating positive, strong, or decisive evidence for the presence of a QTL, respectively.

Moreover, three QTLs were identified in TX2WSE across two environments (years). One QTL on LG4 (*q*BSD.TX2WSE-LG4.1) clustered at 22.14 to 27.27 cM (11.80–20.23 Mbp) with PVE from 7–13%. The other two QTLs were on LG5. *q*BSD.TX2WSE-LG5.1 was detected between 60.51 and 66.26 cM (20.93–24.82 Mbp) with a PVE up to 9% in two different years and locations. The other QTL on LG5, *q*BSD.TX2WSE-LG5.2, was detected twice in 2018 and once in 2020 with wide intervals (74.36–88.70 cM, 35.12–63.28 Mbp) and peaks at 78 and 81 cM with PVE from 9–12%. Two additional QTLs (*q*BSD.TX2WSE-LG1.2 and *q*BSD.TX2WSE-LG4.2) were environment-specific and only detected in one year, whereas the rest of the mapped QTLs appeared only in one month (Table 2 and Fig. 2).

The QTL genotypes at the peak of *q*BSD.TX2WSE-LG3.1 had BSD incidence of 2.93, 1.43, and 0.46 for offspring with the *QQ*, *Qq*, and *qq* QTL genotypes, respectively (Fig. S13B). Generally, the favorable allele (*q*), associated with lower BSD incidence, was less frequent than the unfavorable allele (*Q*) in the germplasm.

### Haplotype, predictive markers, and their sources for important QTLs on LG3

**Figure 3:**
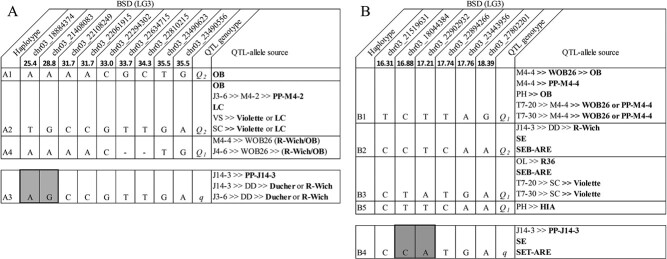
QTL genotypes for black spot on *q*BSD.TX2WOB-LG3.2 for eight diploid rose breeding parents (A) and on *q*BSD.TX2WSE-LG3.1 for nine diploid rose breeding parents (B), with haplotype names, haplotype’s SNP sequences, and origin sources. Alleles for predictive SNP markers associated with *Q-* or *q*-alleles for increasing or decreasing a given trait, respectively, are shaded.

**Figure 4:**
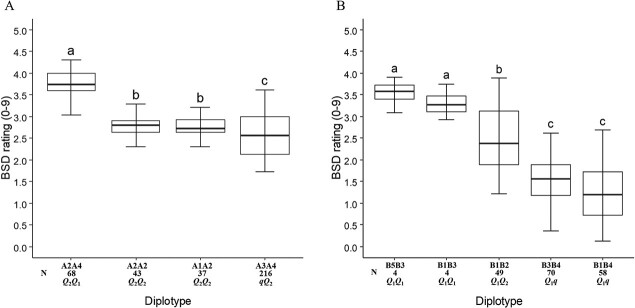
Diplotype effect of the most common haplotypes associated with black spot disease QTL *q*BSD.TX2WOB-LG3.2 (A) and *q*BSD.TX2WSE-LG3.1 (B) in 11 and six diploid rose populations, respectively. Means not connected by the same letter are significantly different (*P < 0.05*) within each population using the nonparametric multiple comparison Steel-Dwass test N = Diplotype sample size

Haplotype analysis was conducted on *q*BSD.TX2WOB-LG3.2 and *q*BSD.TX2WSE-LG3.1. Regarding the TX2WOB population, nine SNP markers between 25.4 and 35.5 cM spanning ~10 cM (~4.6 Mbp) in the *q*BSD.TX2WOB-LG3.2 were selected for haplotype analysis using PediHaplotyper (Fig. 3A). Four distinct SNP haplotypes were identified, of which A1, A2, and A4 were associated with increasing BSD incidence and assigned to the *Q*-allele. A3, the haplotype linked to decreasing the disease incidence, was designated as the *q*-allele (Fig. 3A). A2 was the most prevalent haplotype (Fig. 4A). The non-parametric multiple comparison Steel-Dwass test was used to assess the haplotype/diplotype effect differences. A4 had a greater effect than A2 when comparing the A2A4 to A2A2 diplotypes (Fig. 4A), A1 and A2 had a similar effect based on a comparison of the A2A2 and A1A2 diplotypes. A2 had a greater effect on BSD incidence than A3 by comparing A2A4 to A3A4. Therefore, the haplotype effect size order was A4 > A2 = A1 > A3 corresponding to QTL alleles by *Q_1_, Q_2_*, *Q_2,_* and *q*, respectively. So, this analysis suggests the presence of multiple QTL alleles with different effects. Generally, lower and higher BSD incidence was observed in individuals with the A3A4 (~25% of leaves infected) and A2A4 (~40% of leaves infected) diplotypes, respectively.

A3 (*q*-allele) was differentiated from the other haplotypes (*Q*-alleles) by a pair of adjacent SNP markers, AG-alleles for chr03_18884374 and chr03_21408083 at 25.4 and 28.8 cM (Fig. 3A). Some cultivars and breeding lines in this germplasm shared haplotypes that traced back to various sources. For instance, *Q*_2_ (A2) of five parents was identical-by-state but not identical by descent based on pedigree information since A2 originated from “OB”, PP-M4–2, “Violette”, or “LC”. Likewise, *q* (A3) came from three different sources (PP-J14–3, “Ducher”, or “R-Wich”). On the other hand, *Q_1_* (A4) of M4–4 and J4–6 originated from a recombination event between their founder haplotypes (“R-Wich” and “OB”), whereas “OB” was the only source for A1 (*Q_2_*).

Regarding the TX2WSE population, six SNP markers between 16.31 and 18.39 cM spanning ~2 cM (~6.3 Mbp) were selected in the *q*BSD.TX2WSE-LG3.1 interval for haplotype analysis. Five distinct SNP haplotypes were identified (Fig. 3B). B1, B2, B3, and B5 were associated with increased BSD incidence and were assigned to the *Q*-allele, and B4 was associated with decreased disease incidence and was designated the *q*-allele (Fig. 3B).

The comparison of haplotype/diplotype effects showed that B1 had an equal effect as B5 and B3 when comparing the B5B3 to B1B3 diplotypes and the B3B4 to B1B4 diplotypes, respectively (Fig. 4B). B3 had a greater effect than B2 and B4 by comparing B1B3 to B1B2 and B1B3 to B1B4, respectively. Lastly, B2 significantly increased BSD incidence relative to B4. Hence, the haplotype effects order was B5 = B1 = B3 > B2 > B4, which corresponded to the *Q_1_*, *Q_1_, Q_1_, Q_2_,* and *q* QTL alleles, respectively.

Similar to the analysis of TX2WOB, three functional QTL alleles with different effects were present in this dataset. Overall, B1B4 (~10%) and B5B3 (~35%) conferred more resistance and susceptibility to BSD, respectively (Fig. 4B). In addition, the B4 (*q*-allele) could be differentiated from other haplotypes by a pair of adjacent SNP markers (CA-alleles) at 16.88 cM (18.0 Mb) and 17.21 cM (22.9 Mb) (Fig. 3B).

The pedigree map showed that *Q_2_* (B2) was inherited from R-Wich through J14–3, SE, and SEB-ARE (Fig. 3B). The source of *Q_1_*-allele (B1) came from either “OB” or PPM4–4, while B3 came from R36, SEB-ARE, and “Violette”. B5 was derived from HIA. B4 was traced back to various sources, PP-J14–3, SE, and SET-ARE.

## Discussion

### Narrow sense heritability and G × E interaction

The genetic control of BSD resistance in this germplasm was quantitative and subject to G × E interactions, as has been reported in other studies [9, 18, 19].

BSD resistance showed low to moderate narrow-sense heritability (h^2^) (Table S10 and S11), as previously reported [9, 18]. The very high G × E interaction observed using both datasets was primarily due to the low incidence of BSD in some environments. For instance, GGE biplot illustrates that SV 2021 and CS 2016 discriminated the genotypes differently in the TX2WOB population. Generally, the disease pressure was lower in SV 2019 and SV 2021 than in CS 2016, most probably due to the lower humidity and rainfall during the growing season (460 and 549 mm versus 792 mm) (Table S4) but also due to plant age (Fig. S4) as the inoculum level for BSD increases with the age of the field plot [1]. The same pattern was observed in the TX2WSE population, the high G × E may have resulted from the genotypes responding differently in SV 2018 than in SV 2020 and OV 2019. The SV 2018 showed consistent and high rainfalls and humidity during the evaluation period, and plants were under one year old.

Thus, the high G × E presence in datasets from TX2WOB and TX2WSE populations was anticipated as BSD is a complex trait and greatly influenced by the environment [18, 19, 21]. This illustrates the need to evaluate BSD resistance across different environments, including multiple locations and years, rather than in a single environment. Also, our study highlights the need to account for plant age in field-grown roses. Despite having sensitivity to G × E interactions, quantitative resistance is of interest since it is effective across all pathogenic races and can be more durable than qualitative resistance [11, 18].

### QTL detection

By using PBA under a Bayesian framework, new QTLs and QTLs previously reported for BSD resistance were identified in germplasm from the TAMU rose breeding program. The data analyzed came from two different multi-parental population datasets, planted in three locations and evaluated over 5 years. QTLs were mapped and distributed over all LGs in both populations, except LG7 in the TX2WSE population, indicating the polygenic nature of the resistance to this disease, consistent with previous reports [18–21].

*q*BSD.TX2WOB-LG3.2 (25.4–35.5 cM, 18.8 to 23.4 Mbp) consistently mapped in the same genomic region across five data sets (four in CS 2016 and one in SV 2019) from the TX2WOB population (Table 1 and Fig. 1). This QTL exhibited a PVE of up to 20% and was stable based on the visual inspection of the trace plot. Similarly, in analyses of the TX2WSE population, *q*BSD.TX2WSE-LG3.1 (16.31–18.39 cM, 21.5 to 27.8 Mbp) was also stable and detected across nine environments (three months in each SV 2018, OV 2019, and SV 2020) with PVE of up to 33%. (Table 2 and Fig. 2). This QTL was co-localized with *q*BSD.TX2WOB-LG3.2, however, *q*BSD.TX2WSE-LG3.1 had a wider confidence interval. This may have resulted from the differences in marker density between the two consensus maps used. The TX2WSE ICM had a lower number of markers and less density in LG3 than the TX2WOB ICM (345 vs. 518 markers and 3.7 vs. 6.5 marker/cM) (Table S8 and S9). Furthermore, a region on LG3 with high segregation distortion was noted in this map, which likely corresponds to known self-incompatibility related genes [25]. This result is consistent with earlier findings of the first rose consensus map [15].

These QTLs overlapped with the QTL Meta_1_3 (21.6–24.5 Mbp) [21] and the other QTL on LG3 [22] using diploid rose populations derived from the R-Wich genetic background and multi-year trials. Also, the locations of *q*BSD.TX2WOB-LG3.2 and *q*BSD.TX2WSE-LG3.1 coincide with a partial resistance QTL on LG3 reported by Yan, et al. [19]. This result was expected as this research was conducted on similar genetic materials.

In this work, nine minor QTLs were associated with BSD of which seven of them were novel. The intervals for these QTLs overlapped across data from two environments (years/locations) in this study and/or other earlier studies. Two minor QTLs were identified at different regions on LG1 in analyses of the TX2WOB and TX2WSE populations with PVE up to 11%. The first QTL, *q*BSD.TX2WOB-LG1.1, spanned 12.4 to 22.6 Mbp, was detected in CS 2016 and overlapped with *q*BSD.TX2WSE-LG1.1 (22.6–26.03 Mbp) in SV 2020. However, *q*BSD.TX2WOB-LG1.1 was localized closer to an *Rdr1* homolog in that region [13, 26] than *q*BSD.TX2WSE-LG1.1. The second LG1 QTL was discovered in CS 2016 (*q*BSD.TX2WOB-LG1.2) and SV 2020 (*q*BSD.TX2WSE-LG1.2), spanned 43.80–49.27 Mbp, and was also close to another region with *Rdr1* homologs. *Rdr1* belongs to a large TIR-NBS-LRR (Toll/interleukin-1 receptor-nucleotide binding site-leucine rich repeat) (TNL) gene family that confers resistance to black spot. *Rdr1* homologs have been found in three to four locations in LG 1 with two major clusters at the distal end of LG1 [26].

Two minor QTLs on LG3 were found in different regions in both populations. The first QTL, *q*BSD.TX2WOB-LG3.1 was at the proximal end of LG3, spanned 6.45–11.17 Mbp, and was only seen in SV 2021. While the second LG3 QTL was between 33.83 to 37.81 Mbp was found in SV 2020 (*q*BSD.TX2WSE-LG3.3) and SV 2018 (*q*BSD.TX2WSE-LG3.4) with PVE ranging from 7 to 8%. This finding agreed with Lopez Arias, et al. [21], who found a QTL (Meta_3_2) for BSD at LG3 between 34.22 to 37.77 Mbp. Moreover, five QTLs clustered in LG5 at two different regions were population specific as they were only mapped in TX2WSE. The first LG5 QTL, *q*BSD.TX2WSE-LG5.1, was found in OV 2019 and SV 2020 between 20.93 to 24.82 Mbp and PVE up to 9%. This region was associated with the QTL (Meta_2_5) reported by Lopez Arias, et al. [21] between 18.82 to 24.88 Mbp. The second LG5 QTL, *q*BSD.TX2WSE-LG5.2, was detected in SV 2018 and SV 2020 from 35.12 to 63.28 Mbp with PVE up to 12% and overlapped with the chromosomal position associated with the *Rdr4* gene for black spot resistance (34.11–43.40 Mbp) [14]. Similarly, two minor QTLs were discovered on LG4 between 11.80 to 20.23 Mbp and 25.00 to 40.32 Mbp in TX2WSE. Lastly, the QTL at the proximal end of LG7 (0.20 to 0.59 Mbp) was only observed in the TX2WOB population (CS 2016 and SV 2021) and had a PVE of 15%.

In summary, the variability in some QTL results between the two datasets was expected due to the different consensus maps as well as the high G × E, particularly in the TX2WOB population (${\sigma}_{g\times e}^2/{\sigma}_g^2=10.76$). The PBA approach discovered several new QTLs and confirmed other previously reported QTLs [19–22]. This study confirmed that the chromosomal region on LG3 (*q*BSD.TX2WOB-LG3.2 and *q*BSD.TX2WSE-LG3.1) has the largest effect on resistance to BSD compared to minor effects of other QTLs. This region of LG3 QTL was consistently detected over diverse populations, sample sizes, environments, linkage maps, and QTL mapping approaches. Still, more studies are necessary to validate the new and distinct QTLs identified for BSD using broader and different germplasm with multi-environment trials.

### Haplotype characterization of significant QTLs on LG3

The examination of haplotype/diplotype effects for *q*BSD.TX2WOB-LG3.2 spanning ~4.6 Mbp revealed four distinct haplotypes and three alleles (*Q*_1_ (A4), *Q*_2_ (A1, A2), and *q* (A3). Only A3 was linked to decreasing BSD in TX2WOB (Fig. 3A). This haplotype was derived from PP-J14–3, “Ducher,” or “R-Wich”. A3 is distinguished from other haplotypes (*Q*-alleles) by two pairs of adjacent SNP markers (AG-alleles) at 25.4 and 28.8 cM (18.88 and 21.40 Mbp). A3A4 (*qQ_2_*) was present in about half of the progenies (216) (Fig. 4A). Also, a series of QTL alleles of different effects were discovered and coined *Q*_1_ (A4) and *Q*_2_ (A1 and A2) at this locus.

In the genomic region of *q*BSD.TX2WSE-LG3.1, five haplotypes and three alleles [*Q*_1_ (B1, B3, and B5), *Q*_2_ (B2), and *q* (B4)] were detected. Only B4 was associated with decreasing BSD (*q*-allele) incidence (Fig. 3B). This haplotype originated from PP-J14–3, SE, and SET-ARE. This haplotype was differentiated from B1, B2, B3, and B5 (*Q-alleles*) by two pairs of adjacent SNP markers (CA-alleles) at 16.88 and 17.21 cM (18.0 and 22.9 Mbp). Also, multiple effects associated with increased disease incidence (*Q-allele*) were found and designated the *Q*_1_ (B1, B3, and B5) and *Q*_2_ (B2) QTL genotypes.

This multiallelism found in this study was also described in acidity QTLs in apple and blush, ripening date, and fruit development period in peach (*Prunus persica*) [27–29]. Due to missing genotyping information within the QTL interval, PediHaplotyper failed to identify the haplotype sequence of some individuals in TX2WOB, e.g. one parent of J4–6 and both parents of “RF”.

Overall, the *q*-alleles for both LG3 QTLs in TX2WOB and TX2WSE had one common source, PP-J14–3. Moreover, the physical positions of the two SNP markers associated with *Q*/*q* alleles of both LG3 loci coincided. This finding suggests that qBSD.TX2WOB-LG3.2 and qBSD.TX2WSE-LG3.1 represent the same QTL. Therefore, consistent expression of this QTL across five evaluated years in three locations using different population sets and sample size suggests that this QTL should be used in the genetic enhancement of rose resistance to *D. rosae*.

Ultimately, this research should facilitate the development of a high-throughput predictive DNA test targeting this LG3 QTL region conferring resistance for routine use in the marker-assisted breeding pipeline in rose breeding programs.

### Candidate genes

Hundreds of potential R-genes for disease resistance distributed throughout the *Rosa chinensis* reference genome^25^ were identified by Lopez Arias, et al. [21]. However, the largest number of NB-encoding genes were clustered on the distal ends of chromosomes 1, 5, and 7. Interestingly, the regions where the LG3 QTL and other QTLs were found in this study harbored several candidate R-genes and defense response genes. The interval of LG3 QTL comprised two NBS-LRR genes, the largest group of plant R-genes, and several other NBS-encoding genes. The interval also overlapped with the defense response gene RC3G0142400, which encodes an EMSY-LIKE 1 protein that had a role in Arabidopsis (*Arabidopsis thaliana*) downy mildew resistance [30]. Also, the LG3 QTL interval contains two genes encoding P450 cytochrome, such as CYP736B and CYP72A, which are involved in the defense response against *Xylella fastidiosa* in grapevine (*Vitis*) and *Fusarium graminearum* in wheat (*Triticum*), respectively [31, 32]. Likewise, many NBS-encoding genes were in the interval of minor LG3 QTL (33.83–37.81 Mbp) and co-localized with a transcription factor (RC3G0261500) which had a crucial role in plant immunity [33, 34]. This region also contains the pathogenesis-related thaumatin gene (RC3G0264400) identified for its antifungal activity against filamentous fungi [35, 36].

The region of LG5 QTL (*q*BSD.TX2WSE-LG5.2) encompasses a cluster of ten genes coding for cytochrome P450 and a glucan synthase-like gene (RC5G0249400) that is believed to be engaged in callose formation to respond to fungal attack [37, 38]. Lastly, the QTL on LG1 spanning 43.80 to 49.27 Mbp contains a cluster of NBS-LRR genes and is close to BSD resistance gene *Rdr1*^22^.

### Future work

Additional QTL mapping studies using multi-parental population sets of different germplasm backgrounds with larger family sizes could improve the representation of the three QTL genotype classes and all their diplotype combinations to better characterize these mapped QTLs. Although efforts were made to identify potential candidate genes, with the resources that were available, more studies focusing on the genetic basis of resistance are needed, including more precise localization and fine-mapping of QTLs, identifying and testing more sensible candidate genes, and validating newly detected QTLs. These activities will broaden our understanding of the genetic basis of quantitative resistance of BSD.

## Conclusion

In this study, the pedigree-based QTL mapping software, FlexQTL, identified QTLs (novel and previously reported) distributed over all LGs associated with BSD resistance using two multi-parental populations of diploid rose evaluated over three locations and five years. A total of 13 and 15 QTLs were identified in TX2WOB and TX2WSE populations, respectively. The major QTL on LG3 of both populations was stable and clustered at either 18.8 to 23.4 or 21.5 to 27.8 Mbp, and explained 20 and 33% of the phenotypic variation. These were considered as the same QTL. Furthermore, minor effect QTLs were mapped including two QTLs on LG1 and LG3 (TX2WOB and TX2WSE), two QTLs on LG4 and LG5 (TX2WSE), and one QTL on LG7 (TX2WOB).

The haplotype analysis revealed multiple functional LG3 QTL alleles associated with BSD resistance in the TX2WOB and TX2WSE populations. Unique SNP markers associated with resistance to this fungal disease were identified, and PP-J14–3 was one common source across populations. All mapped QTLs encompassed several putative candidate R-genes and defense response genes. The next step of this work is to convert the SNP haplotypes of resistant alleles into DNA tests (e.g. Kompetitive allele-specific PCR (KASP)) to track and select these factors in a plant breeding context. Use of this knowledge and tools should lead to more effective use of these factors in durable BSD resistance breeding.

## Methods

### Plant materials

BSD field evaluations were conducted on two multi-parental diploid rose populations (TX2WOB and TX2WSE). The 11 F_1_ populations of TX2WOB were generated using a partial diallel design by crossing nine parental lines including well-adapted and black spot resistant breeding lines derived from *R. wichurana* “Basye’s Thornless” (R-Wich), the moderately BSD resistant “Old Blush” (OB), and BSD susceptible cultivars (Fig. S1) [18, 19]. The TX2WOB multi-parental population was evaluated in 2016 and a subset of ten populations of the original were phenotyped in 2019 and 2021 (Table S1).

In 2012, one plant of each member of the TX2WOB population was planted at 1 × 3.5 m spacing in the field at the Horticulture Farm at Texas A&M University in College Station, TX, USA (30.63, −96.37). In 2018, plants of a subset of ten subpopulations of the original TX2WOB population was planted in a randomized complete block design with two replications, where individual plants were the experimental unit. These plants were planted at 1.2 × 1.8 m spacing at the Texas A&M University Horticulture Teaching Research and Extension Center (HortTREC) in Somerville, TX (30.524591, −96.422479). Plants were pruned in the winter (February/March).

The TX2WSE multi-parental population is composed of six F_1_ rose populations from crosses involving nine parents. This population is based on germplasm primarily derived from R-Wich and “Srdce Europy” (SE) (Fig. S2), and has been used to map QTL rose rosette disease resistance [39]. However, the version of TX2WSE multi-parent population used in the present study has fewer subpopulations (six vs. eight) but a larger sample size (378 vs. 248 progenies). The TX2WSE population was evaluated for BSD in 2018, 2019, and 2020 (Table S2). Plants of this population were planted at HortTREC in the spring of 2018 in a completely randomized design with two replications, with individual plants as the experimental units. Plants were planted at 1.2 × 1.2 m spacing. A subset of the subpopulations was also planted in the spring of 2018 with two replications at the Texas A&M AgriLife Research & Extension Center at Overton, TX (32.295920, −94.976125). Plants in the field were arranged by families. The plants were pruned annually in the winter beginning the 2nd year in the field. For weed control, a black fabric weed barrier was used. Plants were regularly irrigated, and no fungicides were applied during the evaluation period.

### Field disease assessment

Briefly, 721 genotypes of the TX2WOB population were evaluated for BSD incidence in College Station (CS), Texas, during June, Sep., Oct., and Nov., 2016 (5^th^ field season). In addition, 218 and 297 progenies were evaluated in Somerville (SV), TX, from June to Nov. in 2019 (2^nd^ field season) and May through Nov. in 2021 (3^rd^ field season), respectively (Table S1). Regarding the TX2WSE population, 378 progenies were evaluated for BSD incidence from Apr. through Nov., 2018 (2^nd^ field season) and May to Aug., 2020 (3^nd^ field season) in SV, TX. Also, 216 progenies were assessed in June and Sep., 2019 (2^nd^ field season) in Overton, TX (Table S2).

Black spot incidence was evaluated by using a percentage-based rating scale of 0 to 9 (0 = no disease symptoms, 1 = 10% of the leaves of the plant canopy showed lesions, 2 = 20%, 3 = 30%, 4 = 40%, 5 = 50%, 6 = 60% of leaves infected, 7 = most foliage infected, 8 = all foliage infected with some defoliation, 9 = all foliage infected, heavy defoliation). Lastly, in all data sets, excluding 2016, least-squares means were estimated using the R package emmeans v. 1.7.5. In turn, corrected means were used in subsequent analyses.

### Heritability and genotype by environment interaction

The Shapiro–Wilk test was to test for normality. This analysis showed that the data were non-normally distributed (*W* ranged from 0.730 to 0.993, *P* < 0.003). Data transformations did not improve normality. Thus, untransformed data was used for variance component analysis. Genetic variance components and heritability were estimated from a linear mixed model using a Restricted Estimated Maximum Likelihood (REML) method in JMP Pro version 13.2 (SAS Institute Inc., Cary, NC. 2016), assuming all effects are random to obtain a more robust analysis for this unbalanced design [40]. The model was:

$$ \begin{align*} y=\mu &+{\sigma}_{FP}^2+{\sigma}_{MP}^2+{\sigma}_{Progeny\left( FP: MP\right)}^2+{\sigma}_{Env}^2+{\sigma}_{FP\ x\ Env}^2+{\sigma}_{MP\ x\ Env}^2\\&+{\sigma}_{Progeny\ x\ Env}^2+{\sigma}_{error}^2 \end{align*}$$

where μ is the BSD incidence mean; σ^2^_FP_ and σ^2^_MP_ are the variances for the female (FP) and male (MP) parent, respectively; σ^2^_Progeny(FP,MP)_ is the variance for progenies of a given cross; σ^2^_Env_ is the variance due to the environment (month/year/location combination); σ^2^_FP × Env_, σ^2^_MP × Env_, and σ^2^_Progeny × Env_ are variances due to the interaction of female and male parents and progenies with the year of assessment; and σ^2^_error_ is the error variance.

The sum of parental variances (σ^2^_FP_ and σ^2^_MP_) was considered as additive variance (${\sigma}_A^2$), progeny variance was treated as non-additive variance (${\sigma}_d^2$), and the sum of the parental and progeny variances was regarded as the genotypic variance (${\sigma}_g^2$). The interaction of genotype [σ^2^_FP_, σ^2^_MP_, and σ^2^_Progeny(FP,MP)_] by environment (month/year/location) was treated as the genetic-environmental variance (${\sigma}_{g\times e}^2$). The residual variance was confounded with progeny × environment variance.

Broad sense heritability across the environments was calculated as:

${H}^2=\frac{\sigma_g^2\ }{\sigma_g^2+\frac{\sigma_{g\times e}^2}{E}}$ where *E* indicates the number of environments [41–44]. The ratio of the genotype by environment variance to the genetic variance was quantified as:

$$ \begin{align*} {\sigma}_{g\times e}^2/\left({\sigma}_g^2\right). \end{align*}$$

A genotype plus genotype-by-environment (GGE) biplot was used to display the genotype by environment interaction (G × E) results using the package “GGEbiplots” (version 0.1.3) of R (version 4.1.2; R Foundation for Statistical Computing, Vienna, Austria). Pearson correlations coefficients were calculated among years.

### SNP genotyping and consensus map construction

Genomic DNA was extracted from young leaves following Doyle’s CTAB protocol [45]. Genotyping by sequencing (GBS) was performed using the restriction enzyme *Ngo*MIV. Single-end sequencing was achieved on an Illumina HiSeq 2500 platform. After trimming the barcodes using a custom python script, the trimmed reads were aligned to the *R. chinensis* v1.0 genome [25] using the CLC Genomics Workbench v9.0 (Qiagen, Boston, MA). After alignment, SNPs were called following the procedures described by Yan, et al. [46].

For the TX2WOB population, 415 individuals from five diploid rose populations were used to construct a consensus map (Table S8). Before map construction, markers mapped to chromosome 0, non-biallelic markers, and markers missing >10% were removed using Tassel version 5. A custom script was used to convert marker calls into allele dosage (nulliplex, simplex, or duplex). Then, the R package polymapR v1.1.1 was employed to develop individual population maps. PolymapR was set to perform further filtration to remove duplicated and distorted (*P* ≥ 0.001) markers.

The consensus map for the TX2WOB population was developed using the R package “LPmerge” version 1.7. Further filtering or thinning steps were performed to decrease the number of markers to reduce the computation time. For instance, only one or two markers at the same genetic position were kept with the priority to keep common markers with less missing data. The lowest root mean square error (RMSE) and the map length, gap, and overall quality were used to determine the best maps. The R package “LinkageMapView” version 2.1.2 and MapChart software version 2.32 were used to visualize the consensus map. SNP markers underwent additional curation in FlexQTL software version 0.1.0.42 to fix or eliminate problematic markers with double recombination or other inheritance conflict issues. Further curation was performed when the other six families were added to the FlexQTL dataset.

For the TX2WSE population, SNPs were called using the CLC Genomics Workbench v11.0.1 and grouped into bins based on their proximity to *Ngo*MIV cut sites in the reference genome. After genotyping, it was determined that the genotype of one parent, “SE”, did not explain progeny genotypes well; thus, the parental genotype was imputed via custom scripts. Population maps for three populations (Table S9) were developed in the R package polymapR v1.1.1. The consensus map for the TX2WSE population was developed with the R package LPmerge and further curation was performed before the QTL analysis as previously described [39].

### QTL mapping and characterization

The pedigree-based QTL analysis was conducted with FlexQTL for each month and the overall mean (over months). The dataset for the TX2WOB population includes phenotypic data collected from three environments (CS 2016, SV 2019, and SV 2021) and 1115 SNP markers. The dataset for the TX2WSE population consists of phenotypic data from three environments (SV 2018, SV 2020, and OV 2019) and 866 SNP markers.

FlexQTL employs a Bayesian approach to infer the number of QTLs by comparing models using posterior estimates through Markov Chain Monte Carlo (MCMC) simulations. First, BSD was analyzed with a mixed model (additive and dominant effects). As a dominant effect was not observed, the analysis was rerun with an additive model at least two times with different parameter settings to ensure reproducibility [27]. MCMC simulations length ranged from 100 000 to 800 000 iterations to store a minimum of 1000 samples with a thinning of 100. The effective sample size (ESS) in the FlexQTL parameter file was set to 101 to ensure sufficient convergence [23]. Other FlexQTL outputs were used to check the QTL stability and the position through posterior and trace plots and the mode of a QTL. Downstream analysis was done in FlexQTL to re-define the QTL region and recalculate additive variances associated with the detected QTL. QTL intervals were identified as a series of successive 2-cM bins with 2lnBF ≥ 2 (Bayes Factor (BF) ≥ 2).

The BF was used to determine the QTL number and position [47], with values greater than 2, 5, and 10 interpreted as positive, strong, and decisive evidence for the presence of QTLs, respectively. Thus, in this study, major QTLs include those detected in two or more environments (year/location) with strong (BF ≥ 5) or decisive evidence (BF ≥ 10), having overlapping intervals, and explaining at least 10% of the phenotypic variation.

From FlexQTL outputs, the additive variance (${\sigma}_{A(trt)}^2$) was calculated by subtracting the residual variance $\big({\sigma}_e^2\big)$ from the phenotypic variance $\big({\sigma}_P^2\big)$ for a trait. The narrow-sense heritability (h^2^) for a trait was calculated using the following equation:

$$ \begin{align*} {h}^2=\frac{\sigma_{A(trt)}^2}{\sigma_P^2} \end{align*}$$

The phenotypic variance explained (PVE) by a QTL is calculated using the following equation:

$$ \begin{align*}{PVE}_{\mathrm{additive}\ \mathrm{model}}=\frac{\sigma_{A(qtl)}^2}{\sigma_P^2}\times 100 \end{align*}$$

where:

${\sigma}_{A(qtl)}^2$ : additive variance of QTL

QTLs were named according to Genome Database for Rosaceae guidelines [48]. Thus, in the name *q*BSD*.*TX2WOB-LG3.1, *q* stands for QTL, “BSD” stands for the black spot disease, “TX2WOB” or “TX2WSE” the name of consensus maps (based on the multi-parental populations used to construct them), “LG3” the linkage group number, “1” or “2” to distinguish different QTLs in case there is more than one QTL on the same linkage group.

Haplotyping was conducted for SNPs within the interval of a significant QTL with strong or decisive evidence and consistently showed high PVE. Haplotypes were constructed across the germplasm using FlexQTL and “PediHaplotyper” package of R [49]. Haplotype effects were analyzed manually to examine for the presence of multi-allelic QTLs. Haplotype effects were inferred from combinations of diplotypes. For example, by comparing the effects of the H3|H1 and H3|H2 diplotypes, the effects of H1 and H2 could be determined. A nonparametric multiple comparison Steel-Dwass test (*P* < 0.05) using JMP Pro version 13.2 (SAS Institute Inc., Cary, NC. 2016) was used to assess diplotype effect differences. QTL allele categories (*Q* or *q*) were assigned to haplotypes based on whether their effects increased or decreased disease. *Q*- and *q-*alleles were distinguished by an index number if there was a multi-allelic series. The source of alleles associated with increased or decreased disease incidence was traced back to the furthest ancestor using pedigree records [28, 29].

## Acknowledgments

This work was supported by the Robert E. Basye Endowment in Rose Genetics and the US Department of Agriculture Specialty Crop Research Initiative projects “Combating rose rosette disease: short term and long-term approaches” (2014-51181-22644); “RosBREED: combining disease resistance with horticultural quality in new rosaceous cultivars” (2014-51181-22378); and “Tools for genomics-assisted breeding polyploids: development of a community resource” (2020-51181-32156). We also acknowledge the Turkish Ministry of National Education’s funding provided to S.N.

The authors wish to thank Natalie Anderson and Pamela Hornby (Texas A&M University) for population maintenance. Special thanks to Natalie Patterson (Texas A&M University) for helping with lab techniques, Nolan Bentley (University of Texas, Austin) and Jeekin Lau (Texas A&M University) for help with scripts, and Eric van de Weg (Wageningen University) for training and advice on the use of FlexQTL.

## Author contributions

D.H.B. conceived this study, Z.J.R. and M.Y.R. carried out analyses, E.L.Y., M.Y., S.N., T.H., and Q.F. collected and provided phenotypic data, M.Y. and E.L.Y. collected tissues and extracted DNA, P.E.K. conducted genotyping-by-sequencing and SNP calling, Z.J.R. and E.L.Y. curated genotypic data and produced the linkage maps, Z.J.R. wrote the original draft, Z.J.R., E.L.Y., M.Y., S.N., Q.F., T.H., M.Y.R. P.E.K., D.H.B, and O.R.L. reviewed and edited the manuscript, D.H.B. and O.R.L. provided project supervision. All authors read and approved the final manuscript.

## Data availability

Datasets supporting this study will be available in the Genome Database for Rosaceae (http://www.rosaceae.org).

## Conflict of interests

The authors declare there are no conflicts of interest.

## Supplementary data

Supplementary data is available at *Horticulture Research * online.

## Supplementary Material

Web_Material_uhac183

## References

[1] Debener T , Byrne DH. Disease resistance breeding in rose: current status and potential of biotechnological tools. *Plant Sci*. 2014;228:107–17. 25438791 10.1016/j.plantsci.2014.04.005

[2] USDA/NASS . 2012 Census of agriculture: census of horticultural specialties (2014). AC-12-SS-3. Vol. 3. Washington D.C.: USDA/NASS, 2015. 27.

[3] USDA/NASS . 2017 Census of agriculture: census of horticultural specialties (2019). AC-17-SS-3. Vol. 3. Washington D.C.: USDA/NASS, 2020. 27.

[4] Horst RK , Cloyd RA. Infectious diseases: diseases caused by fungi. In: Compendium of rose diseases and pests, 2nd edn. St. Paul, MN: APS Press, 2007, 8–12.

[5] Byrne DH , Pemberton HB, Holeman DJ et al. Survey of the rose community: desired rose traits and research issues. In: Acta Hortic. 1232nd ed. International Society for Horticultural Science (ISHS): Leuven, Belgium, 2019, 189–92.

[6] Walker S , Mandegaran Z, Roberts AM. Screening roses for resistance to *Diplocarpon rosae*. In: Morisot A, Ricci P, eds. Acta Hortic. Vol. 424. International Society for Horticultural Science (ISHS): Leuven, Belgium, 1996,209–14.

[7] Reddy S , Spencer JA, Newman SE. Leaflet surfaces of blackspot-resistant and susceptible roses and their reactions to fungal invasion. *HortScience*. 1992;27:133–5.

[8] Byrne DH . Advances in rose breeding and genetics in North America. In: Acta Hortic. 1064th ed. International Society for Horticultural Science (ISHS): Leuven, Belgium, 2015,89–98.

[9] Whitaker VM , Zuzek K, Hokanson SC. Resistance of 12 rose genotypes to 14 isolates of *Diplocarpon rosae* wolf (rose blackspot) collected from eastern North America. *Plant Breed*. 2007;126:83–8.

[10] Debener T . Genetics | Inheritance of characteristics. In: Roberts AV (ed.), Encyclopedia of Rose Science. London, UK: Elsevier, 2003,286–92.

[11] Whitaker VM , Bradeen JM, Debener T et al. Rdr3, a novel locus conferring black spot disease resistance in tetraploid rose: genetic analysis, LRR profiling, and SCAR marker development. *Theor Appl Genet*. 2010;120:573–85. 19847388 10.1007/s00122-009-1177-0

[12] Hattendorf A , Linde M, Mattiesch L et al. Genetic analysis of rose resistance genes and their localisation in the rose genome. In: Acta Hortic. 651st ed. (International Society for Horticultural Science (ISHS): Leuven, Belgium, 2004,123–30.

[13] von Malek B , Weber WE, Debener T. Identification of molecular markers linked to Rdr1, a gene conferring resistance to blackspot in roses. *Theor Appl Genet*. 2000;101:977–83.

[14] Zurn JD , Zlesak DC, Holen M et al. Mapping a novel black spot resistance locus in the climbing rose Brite eyes™ (‘RADbrite’). *Front Plant Sci*. 2018;9:1730. 10.3389/fpls.2018.01730 PMC6275305 30534133

[15] Spiller M , Linde M, Hibrand-Saint Oyant L et al. Towards a unified genetic map for diploid roses. *Theor Appl Genet*. 2011;122:489–500. 20936462 10.1007/s00122-010-1463-x

[16] Zurn JD , Zlesak DC, Holen M et al. Mapping the black spot resistance locus *Rdr3* in the shrub rose ‘George Vancouver’ allows for the development of improved diagnostic markers for DNA-informed breeding. *Theor Appl Genet*. 133:2011–20, 2020. 10.1007/s00122-020-03574-4 32166372

[17] Whitaker VM , Hokanson SC. Partial resistance to black spot disease in diploid and tetraploid roses: general combining ability and implications for breeding and selection. *Euphytica*. 2009;169:421–9.

[18] Dong Q , Wang X, Byrne DH et al. Characterization of partial resistance to black spot disease of *Rosa* sp. *horts*. 2017;52:49–53.

[19] Yan M , Byrne DH, Klein PE et al. Black spot partial resistance in diploid roses: QTL discovery and linkage map creation. In: Acta Hortic. 1232nd ed. International Society for Horticultural Science (ISHS): Leuven, Belgium, 2019,135–42.

[20] Soufflet-Freslon V , Marolleau B, Thouroude T et al. Development of tools to study rose resistance to black spot. In: Acta Hortic. 1232nd ed. International Society for Horticultural Science (ISHS): Leuven, Belgium, 2019,213–20.

[21] Lopez Arias DC , Chastellier A, Thouroude T et al. Characterization of black spot resistance in diploid roses with QTL detection, meta-analysis and candidate-gene identification. *Theor Appl Genet*. 2020;133:3299–321. 32844252 10.1007/s00122-020-03670-5

[22] Lopez Arias DC , Chastellier A, Thouroude T et al. High density SNP and SSR linkage map and QTL analysis for resistance to black spot in segregating rose population. In: Acta Hortic. 1283rd ed. International Society for Horticultural Science (ISHS): Leuven, Belgium, 2020,191–8.

[23] Bink MCAM , Jansen J, Madduri M et al. Bayesian QTL analyses using pedigreed families of an outcrossing species, with application to fruit firmness in apple. *Theor Appl Genet*. 2014;127:1073–90. 24567047 10.1007/s00122-014-2281-3

[24] Bourke PM , van Geest G, Voorrips RE et al. polymapR—linkage analysis and genetic map construction from F_1_ populations of outcrossing polyploids. *Bioinformatics*. 2018;34:3496–502. 29722786 10.1093/bioinformatics/bty371 PMC6184683

[25] Hibrand Saint-Oyant L , Ruttink T, Hamama L et al. A high-quality genome sequence of *Rosa chinensis* to elucidate ornamental traits. *Nature Plants*. 2018;4:473–84. 29892093 10.1038/s41477-018-0166-1 PMC6786968

[26] Menz I , Lakhwani D, Clotault J et al. Analysis of the *Rdr1* gene family in different Rosaceae genomes reveals an origin of an R-gene cluster after the split of Rubeae within the Rosoideae subfamily. *PLoS One*. 2020;15:e0227428. 31971947 10.1371/journal.pone.0227428 PMC6977733

[27] Verma S , Evans K, Guan Y et al. Two large-effect QTLs, *ma* and *Ma3*, determine genetic potential for acidity in apple fruit: breeding insights from a multi-family study. *Tree Genet Genomes*. 2019;15:18.

[28] Rawandoozi ZJ , Hartmann TP, Carpenedo S et al. Identification and characterization of QTLs for fruit quality traits in peach through a multi-family approach. *BMC Genomics*. 2020;21:522. 32727362 10.1186/s12864-020-06927-x PMC7392839

[29] Rawandoozi ZJ , Hartmann TP, Carpenedo S et al. Mapping and characterization QTLs for phenological traits in seven pedigree-connected peach families. *BMC Genomics*. 2021;22:187. 33726679 10.1186/s12864-021-07483-8 PMC7962356

[30] Tsuchiya T , Eulgem T. EMSY-like genes are required for full RPP7-mediated race-specific immunity and basal defense in arabidopsis. *Mol Plant-Microbe Interact*. 2011;24:1573–81. 21830950 10.1094/MPMI-05-11-0123

[31] Schuler MA , Werck-Reichhart D. Functional genomics of P450S. *Annu Rev Plant Biol*. 2003;54:629–67. 14503006 10.1146/annurev.arplant.54.031902.134840

[32] Schuler MA , Duan H, Bilgin M et al. Arabidopsis cytochrome P450s through the looking glass: a window on plant biochemistry. *Phytochem Rev*. 2006;5:205–37.

[33] Lui S , Luo C, Zhu L et al. Identification and expression analysis of WRKY transcription factor genes in response to fungal pathogen and hormone treatments in apple (*Malus domestica*). *J Plant Biol*. 2017;60:215–30.

[34] Yang B , Jiang Y, Rahman MH et al. Identification and expression analysis of WRKY transcription factor genes in canola (*Brassica napus* L.) in response to fungal pathogens and hormone treatments. *BMC Plant Biol*. 2009;9:68. 19493335 10.1186/1471-2229-9-68 PMC2698848

[35] Singh NK , Kumar KRR, Kumar D et al. Characterization of a pathogen induced thaumatin-like protein gene *AdTLP* from *Arachis diogoi*, a wild peanut. *PLoS One*. 2013;8:e83963. 24367621 10.1371/journal.pone.0083963 PMC3868660

[36] Zhang J , Wang F, Liang F et al. Functional analysis of a pathogenesis-related thaumatin-like protein gene *TaLr35PR5* from wheat induced by leaf rust fungus. *BMC Plant Biol*. 2018;18:76. 29728059 10.1186/s12870-018-1297-2 PMC5935958

[37] Voigt CA . Cellulose/callose glucan networks: the key to powdery mildew resistance in plants? *New Phytol*. 2016;212:303–5. 27641960 10.1111/nph.14198

[38] Voigt CA . Callose-mediated resistance to pathogenic intruders in plant defense-related papillae. *Front Plant Sci*. 2014;5:168. 10.3389/fpls.2014.00168 PMC4009422 24808903

[39] Young EL , Lau J, Bentley NB et al. Identification of QTLs for reduced susceptibility to rose rosette disease in diploid roses. *Pathogens*. 2022;11:660. 10.3390/pathogens11060660 PMC9227826 35745514

[40] Ramon CL , Milliken GA, Stroup WW, Wolfinger RD. SAS System for Mixed Models. Cary, NC: SAS Institute, Inc., 1996.

[41] Holland JB , Nyquist WE, Cervantes-Martínez CT. Estimating and Interpreting Heritability for Plant Breeding: An Update. In: Janick J, ed. Plant Breeding Reviews Vol. 22. John Wiley & Sons, Inc.: Oxford, UK, 2010,9–112 Ch. 2.

[42] Liang S , Wu X, Byrne D. Genetic analysis of flower size and production in diploid rose. *J Amer Soc Hort Sci*. 2017;142:306–13.

[43] Wu X , Liang S, Byrne DH. Heritability of plant architecture in diploid roses (*Rosa* spp.). *HortScience*. 2019;54:236–9.

[44] Rawandoozi Z , Hartmann T, Byrne D et al. Correlation, and genotype by environment interaction of Phenological and fruit quality traits in peach. *J Amer Soc Hort Sci*. 2021;146:56–67.

[45] Doyle J , Doyle J. DNA isolation from small amounts of plant tissue. *Phytochemical bulletin*. 1991;57:13–5.

[46] Yan M , Byrne DH, Klein PE et al. Genotyping-by-sequencing application on diploid rose and a resulting high-density SNP-based consensus map. *Hortic Res*. 2018;5:17. 29619228 10.1038/s41438-018-0021-6 PMC5878828

[47] Kass RE , Raftery AE. Bayes factors. *J Amer Stat Assn*. 1995;90:773–95.

[48] Jung S , Lee T, Cheng CH et al. 15 years of GDR: new data and functionality in the genome database for Rosaceae. *Nucleic Acids Res*. 2019;47:D1137–d1145. 30357347 10.1093/nar/gky1000 PMC6324069

[49] Voorrips RE , Bink MCAM, Kruisselbrink JW et al. PediHaplotyper: software for consistent assignment of marker haplotypes in pedigrees. *Mol Breeding*. 2016;36:119. 10.1007/s11032-016-0539-y PMC4977329 27547106

