# Correction to: QTL mapping and characterization of black spot disease resistance using two multi-parental diploid rose populations

**DOI:** 10.1093/hr/uhad059

**Published:** 2022-08-25

This is a correction to: Zena J Rawandoozi, Ellen L Young, Muqing Yan, Seza Noyan, Qiuyi Fu, Tessa Hochhaus, Maad Y Rawandoozi, Patricia E Klein, David H Byrne, Oscar Riera-Lizarazu, QTL mapping and characterization of black spot disease resistance using two multi-parental diploid rose populations, Horticulture Research, Volume 9, 2022, uhac183, https://doi.org/10.1093/hr/uhac183

The originally published version of this manuscript contained several errors, which have now been corrected.

On page 3, column 1, line 28, ‘25’ has been corrected to ‘26’ in the following sentence:

In the analysis of data from TX2WOB, one major QTL was discovered consistently on LG3 (qBSD.TX2WOB-LG3.2) over five environments in 2016 and 2019 with positive, strong, and decisive evidence and high posterior intensity (Table S10, Fig. S11, and S12). qBSD.TX2WOB-LG3.2 was localized to an interval between 25.4 and 35.5 cM (peaks 25, 29, 30, 32, and 35 cM), and 18.8 and 23.4 Mbp on the rose genome (Table 1 and Fig. 1).

On page 4, in Table 1, the heading ‘Interval’ was not centered between columns 6 (cM) and 7 (Mbp).

On page 4, in Table 1, in column 5, row 12, value ‘25’ was erroneous and has now been corrected to ‘26’.

On page 4, in Table 1, in column 5, row 15, value ‘75’ was erroneous and has now been corrected to ‘76’.

On page 5, in Table 2, ‘(cM)’ was omitted from the subheading for column ’Mode’.

On page 5, in Table 2, the heading ‘Interval’ was not centered between columns 6 and 7. In addition, column was 7 incorrectly given as (cM), instead of (Mbp).

On page 5, in Table 2, in column 5, row 22, value ‘32’ was erroneous and has now been corrected to ‘33’.

In Figure 1, the population name ‘TX2Blsp1’ in QTL names was incorrect and has been corrected to ‘TX2WOB’.

On page 9, in column 2, line 4, ‘A3A4 (qQ2)’ has been corrected to ‘A3A4 (qQ1)’.

