# Supplementary figures and images for: QTL mapping and characterization of black spot disease resistance using two multi-parental diploid rose populations
Source: Hortic Res. 2022 Aug 25;9:uhac183. doi: 10.1093/hr/uhac183 (PMC10101596; doi:10.1093/hr/uhac183)

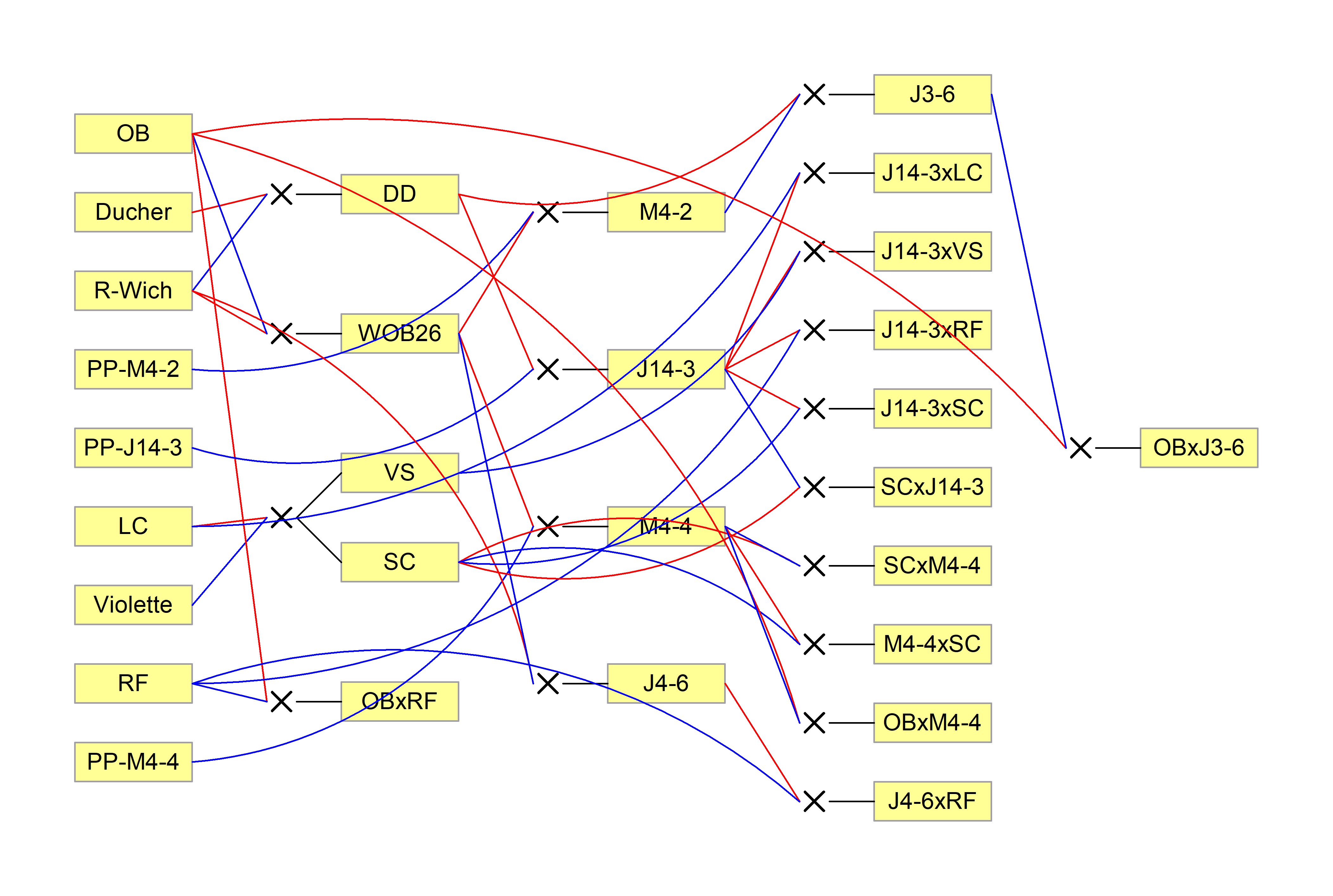

Supplement: Web_Material_uhac183 [file web_material_uhac183.zip › Fig. S1.jpg]

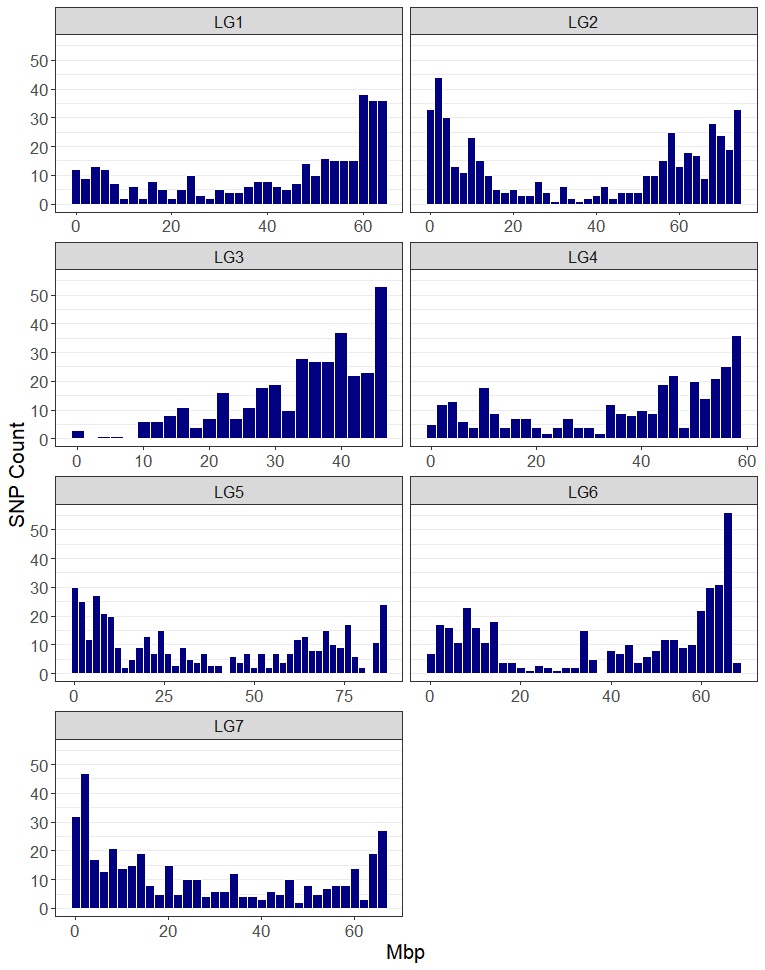

Supplement: Web_Material_uhac183 [file web_material_uhac183.zip › Fig. S10.jpg]

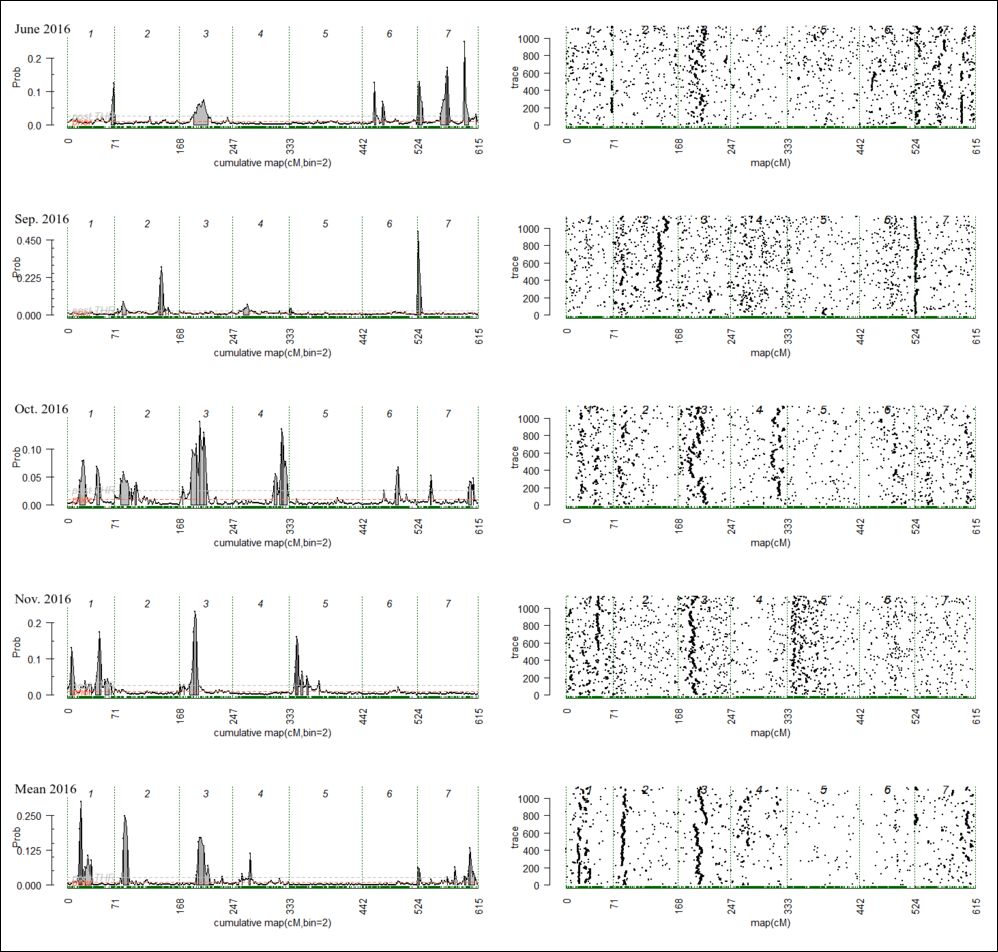

Supplement: Web_Material_uhac183 [file web_material_uhac183.zip › Fig. S11.jpg]

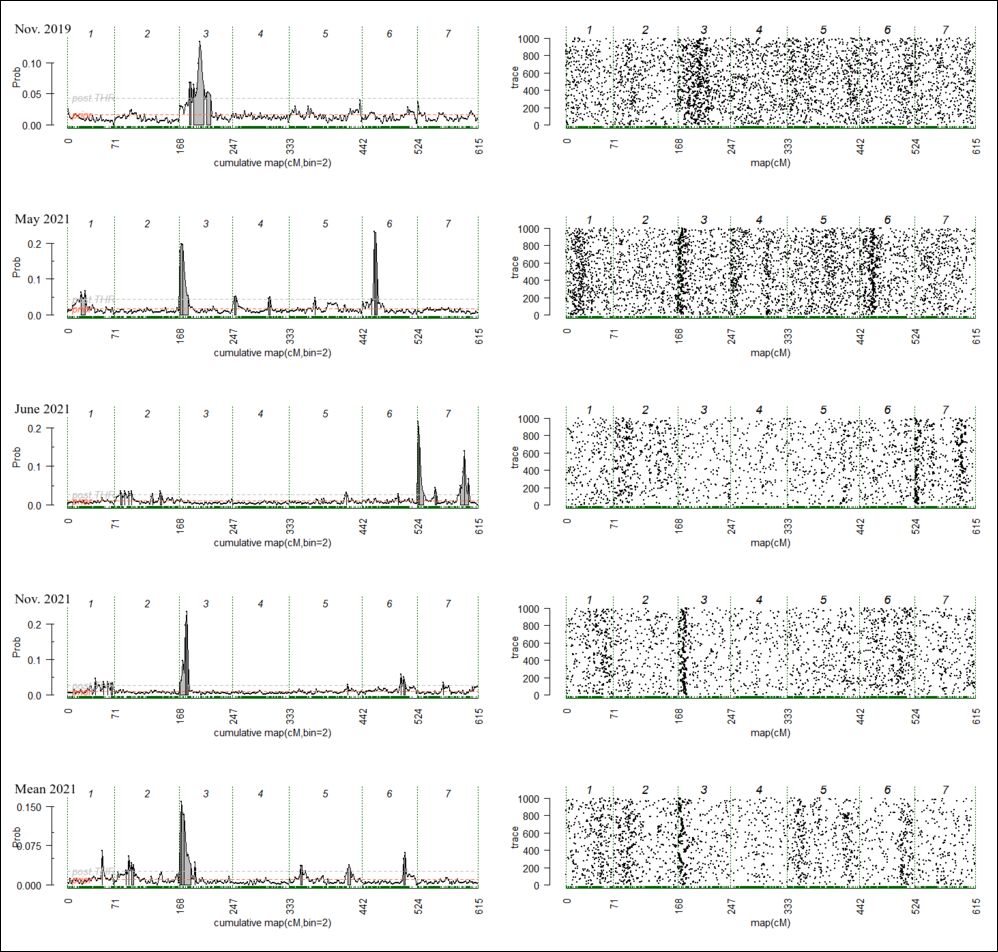

Supplement: Web_Material_uhac183 [file web_material_uhac183.zip › Fig. S12.jpg]

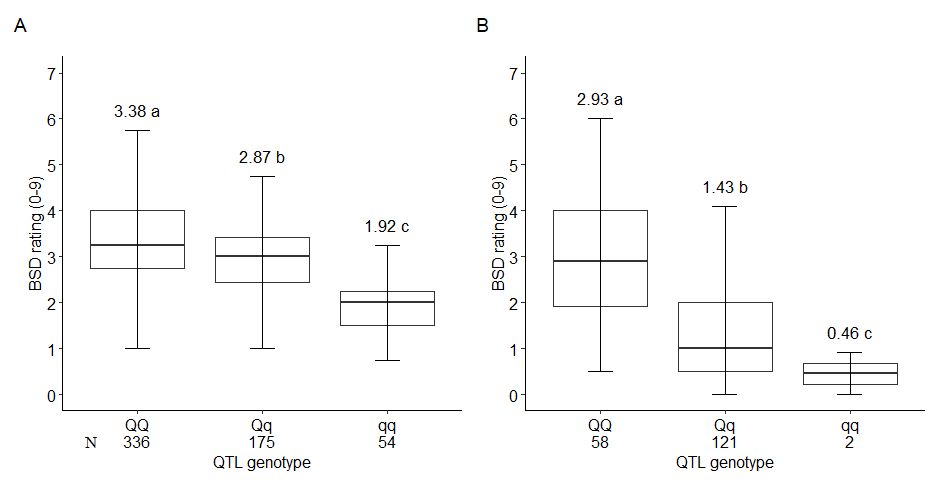

Supplement: Web_Material_uhac183 [file web_material_uhac183.zip › Fig. S13.jpg]

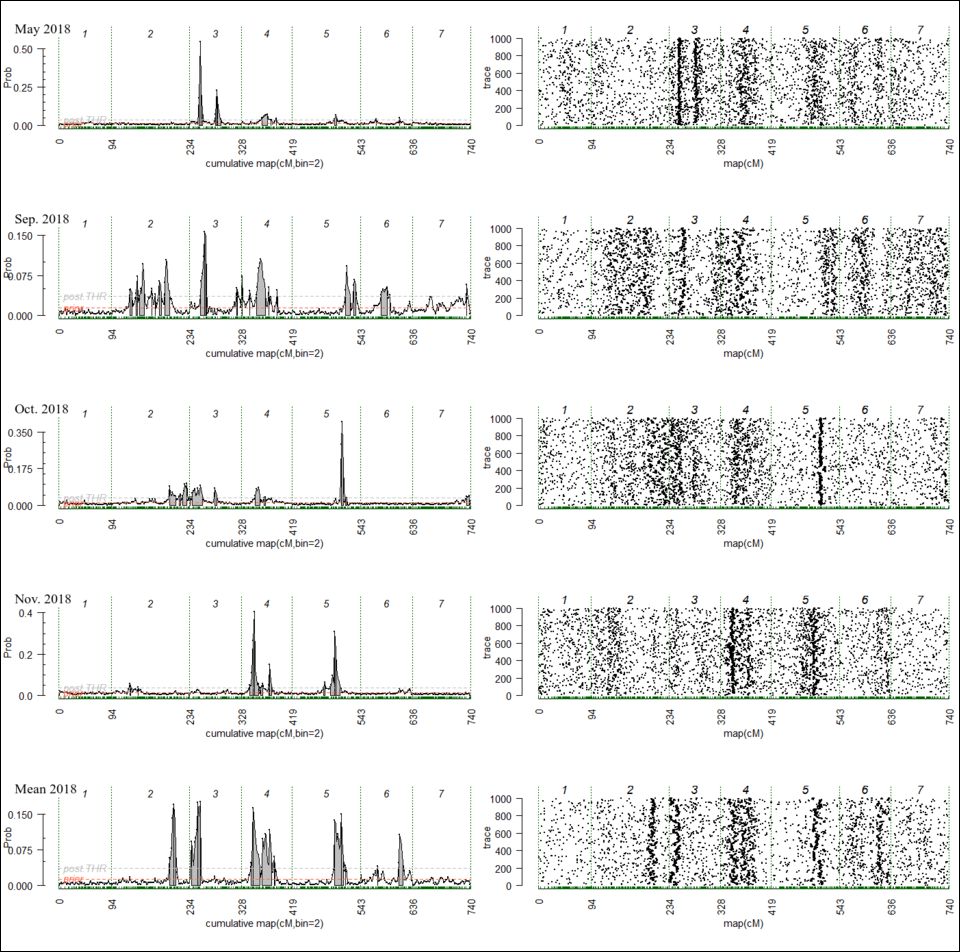

Supplement: Web_Material_uhac183 [file web_material_uhac183.zip › Fig. S14.jpg]

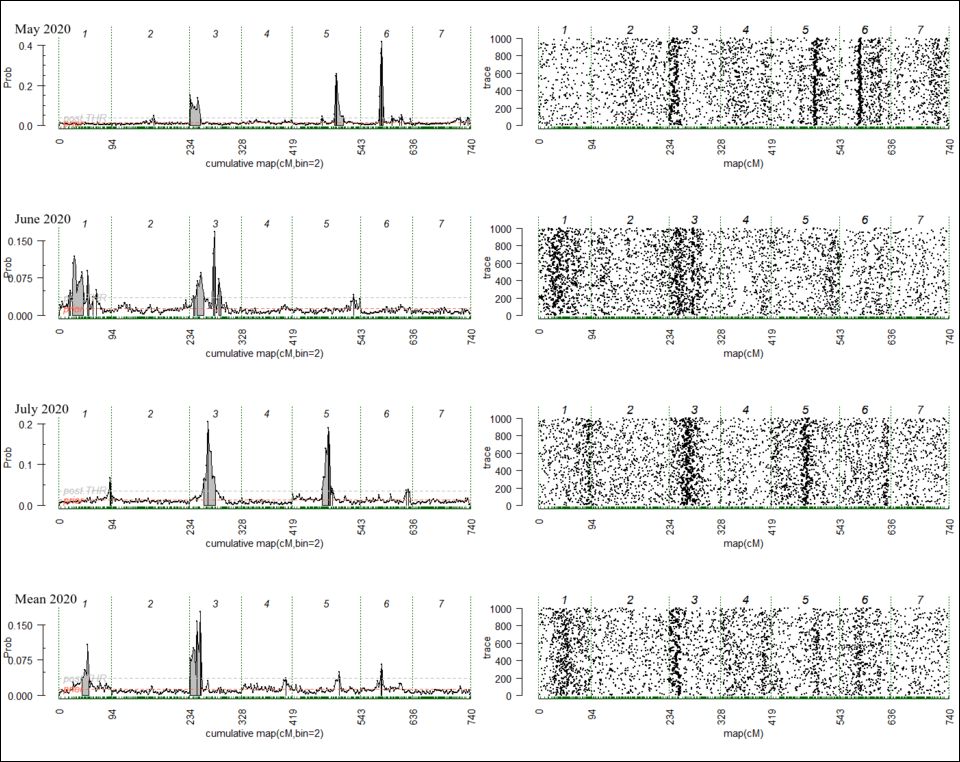

Supplement: Web_Material_uhac183 [file web_material_uhac183.zip › Fig. S15.jpg]

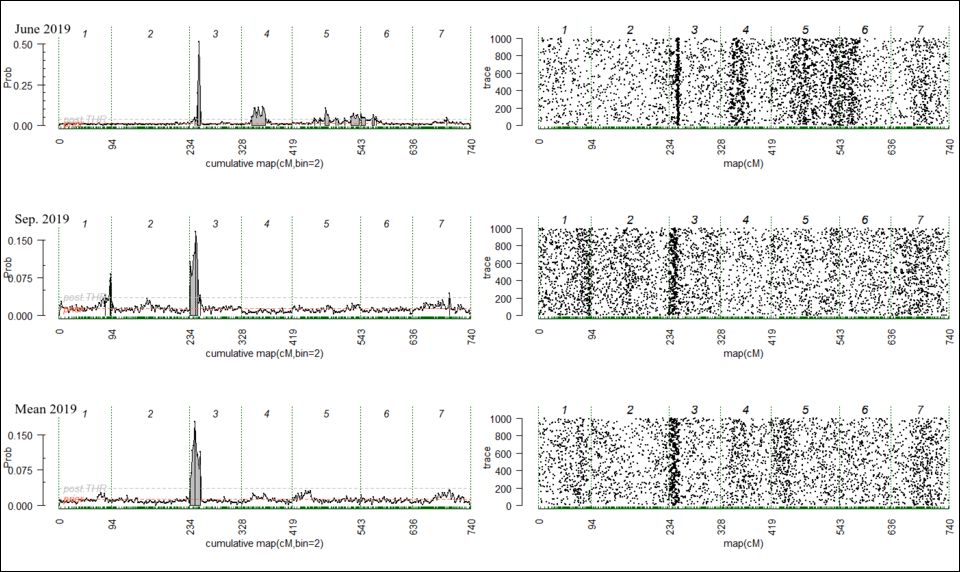

Supplement: Web_Material_uhac183 [file web_material_uhac183.zip › Fig. S16.jpg]

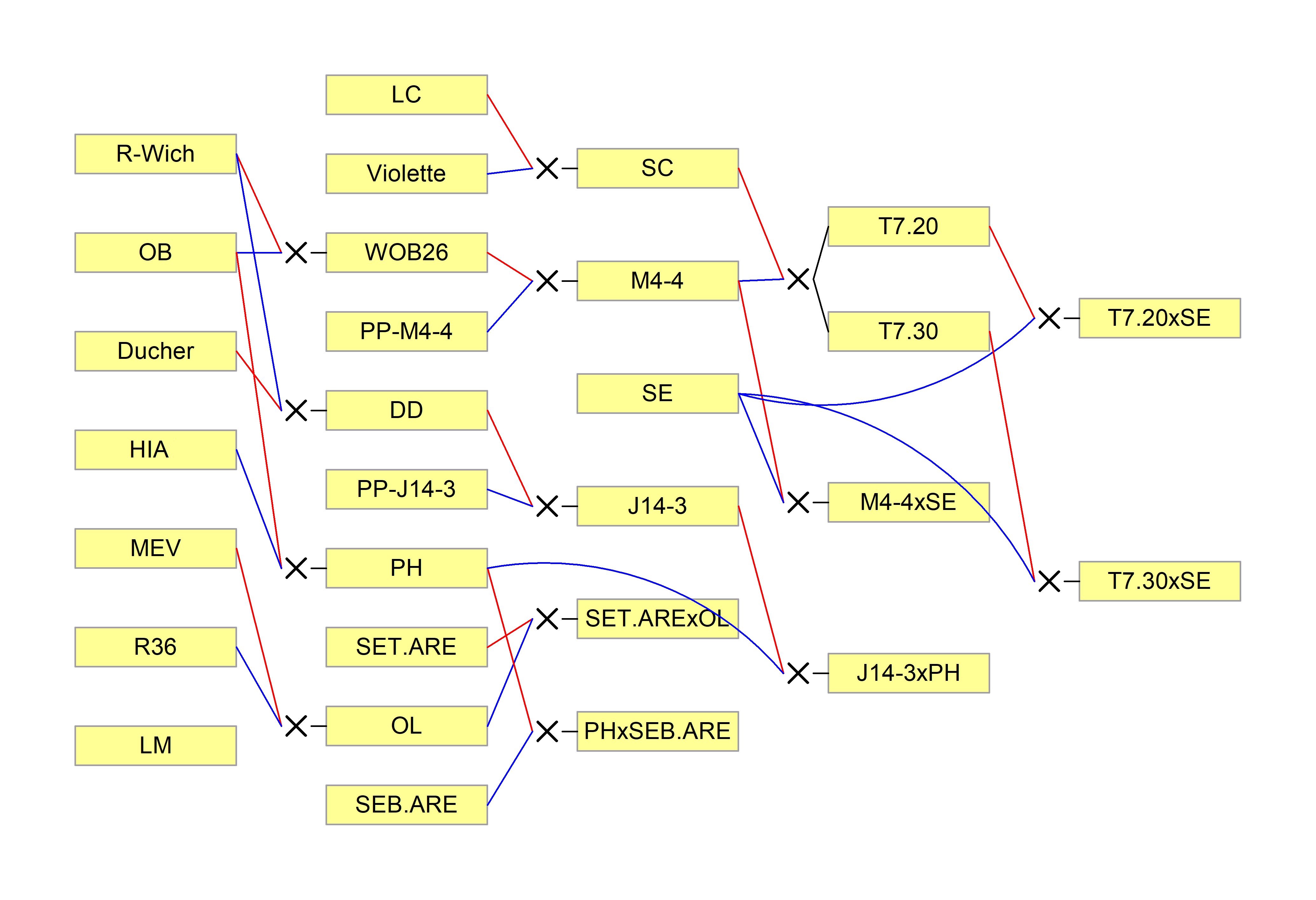

Supplement: Web_Material_uhac183 [file web_material_uhac183.zip › Fig. S2.jpg]

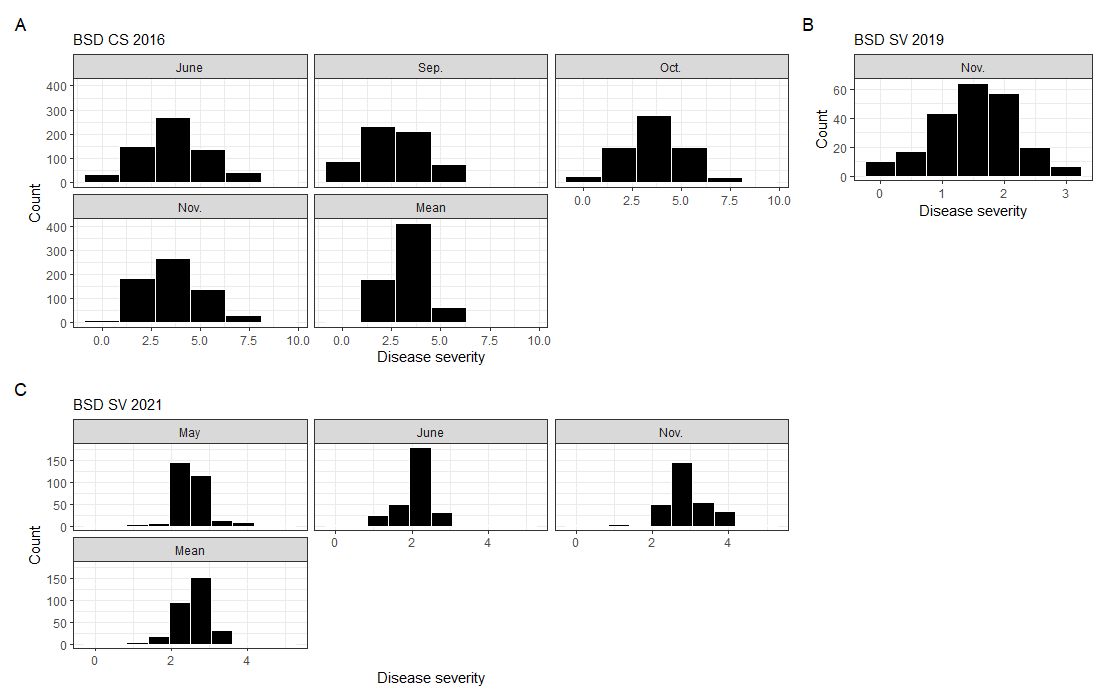

Supplement: Web_Material_uhac183 [file web_material_uhac183.zip › Fig. S3.jpg]

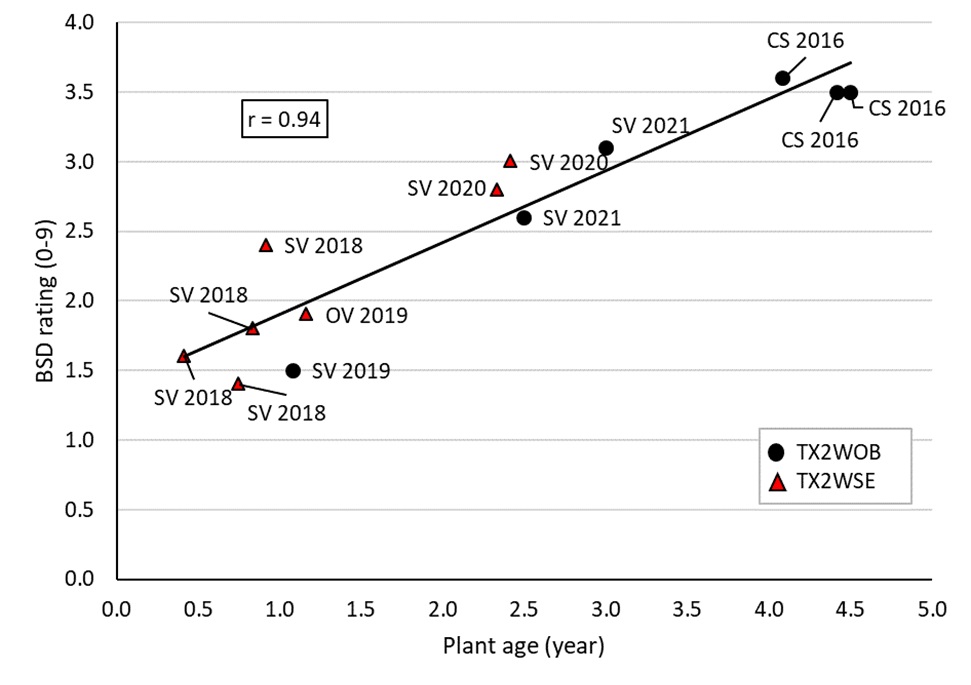

Supplement: Web_Material_uhac183 [file web_material_uhac183.zip › Fig. S4.jpg]

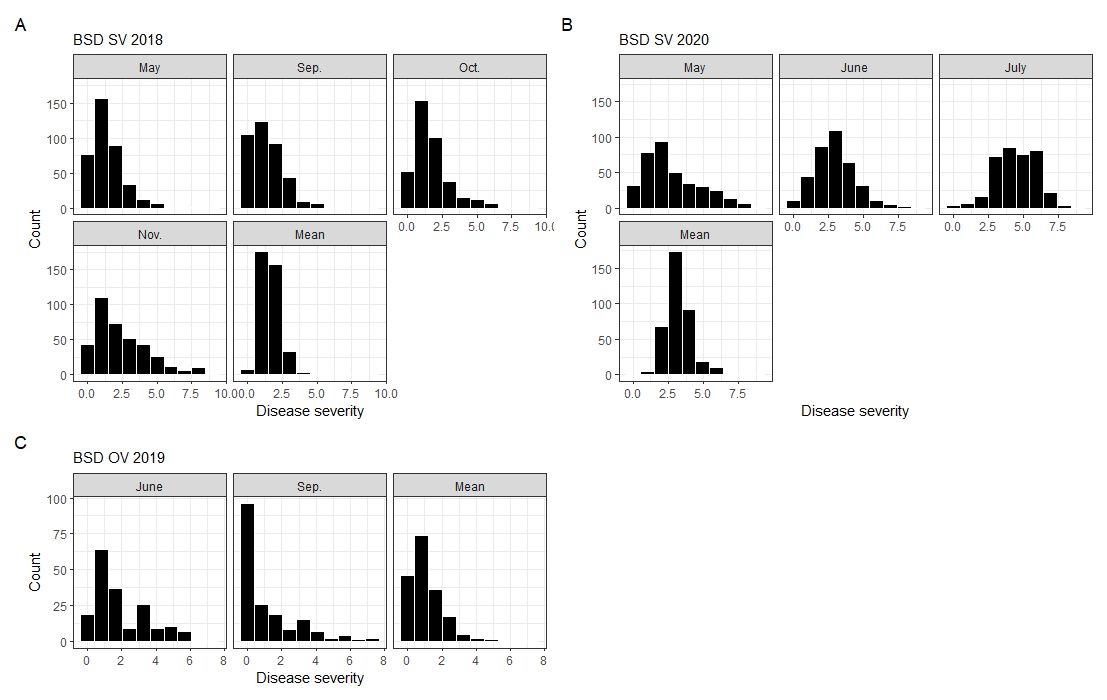

Supplement: Web_Material_uhac183 [file web_material_uhac183.zip › Fig. S5.jpg]

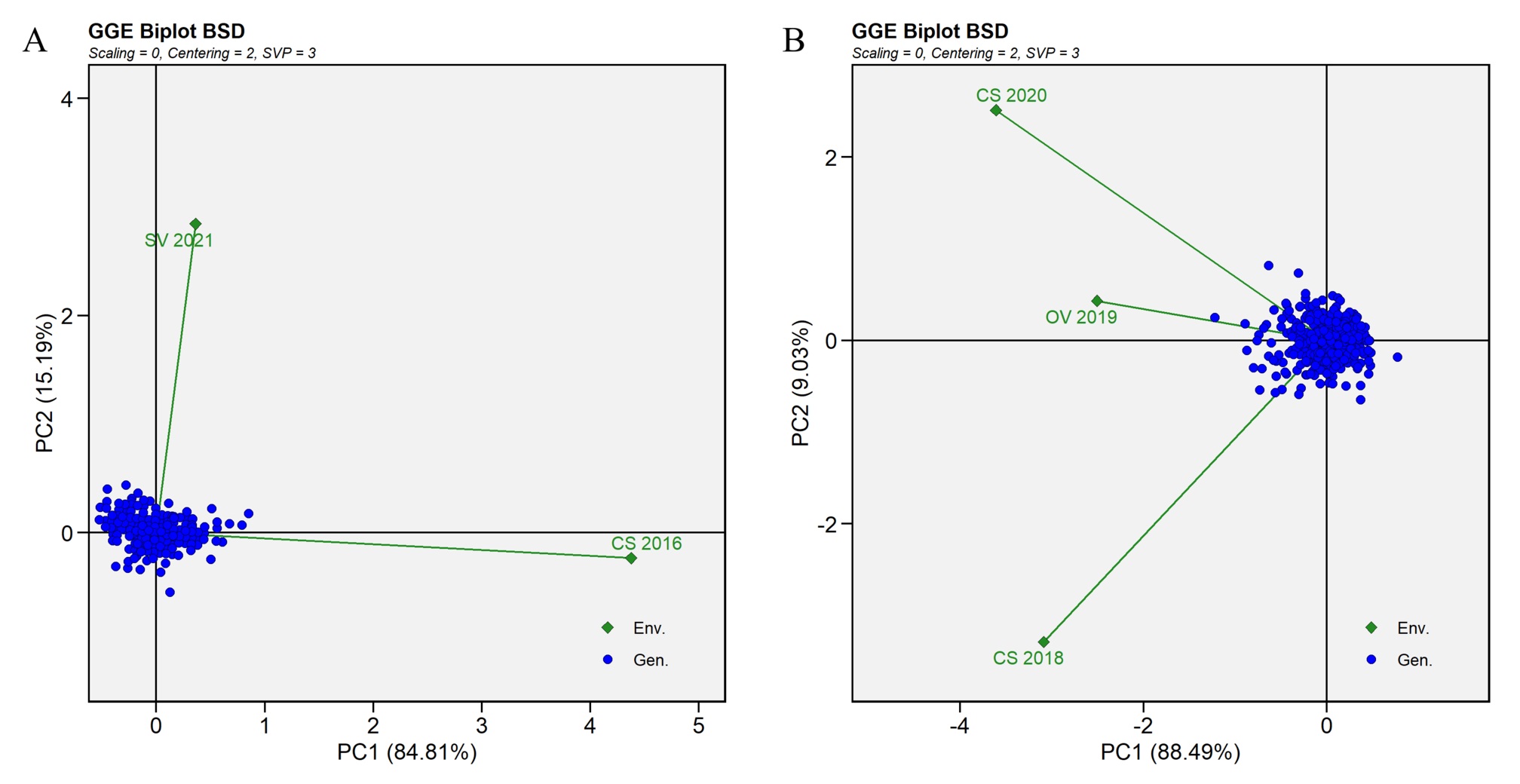

Supplement: Web_Material_uhac183 [file web_material_uhac183.zip › Fig. S6.jpg]

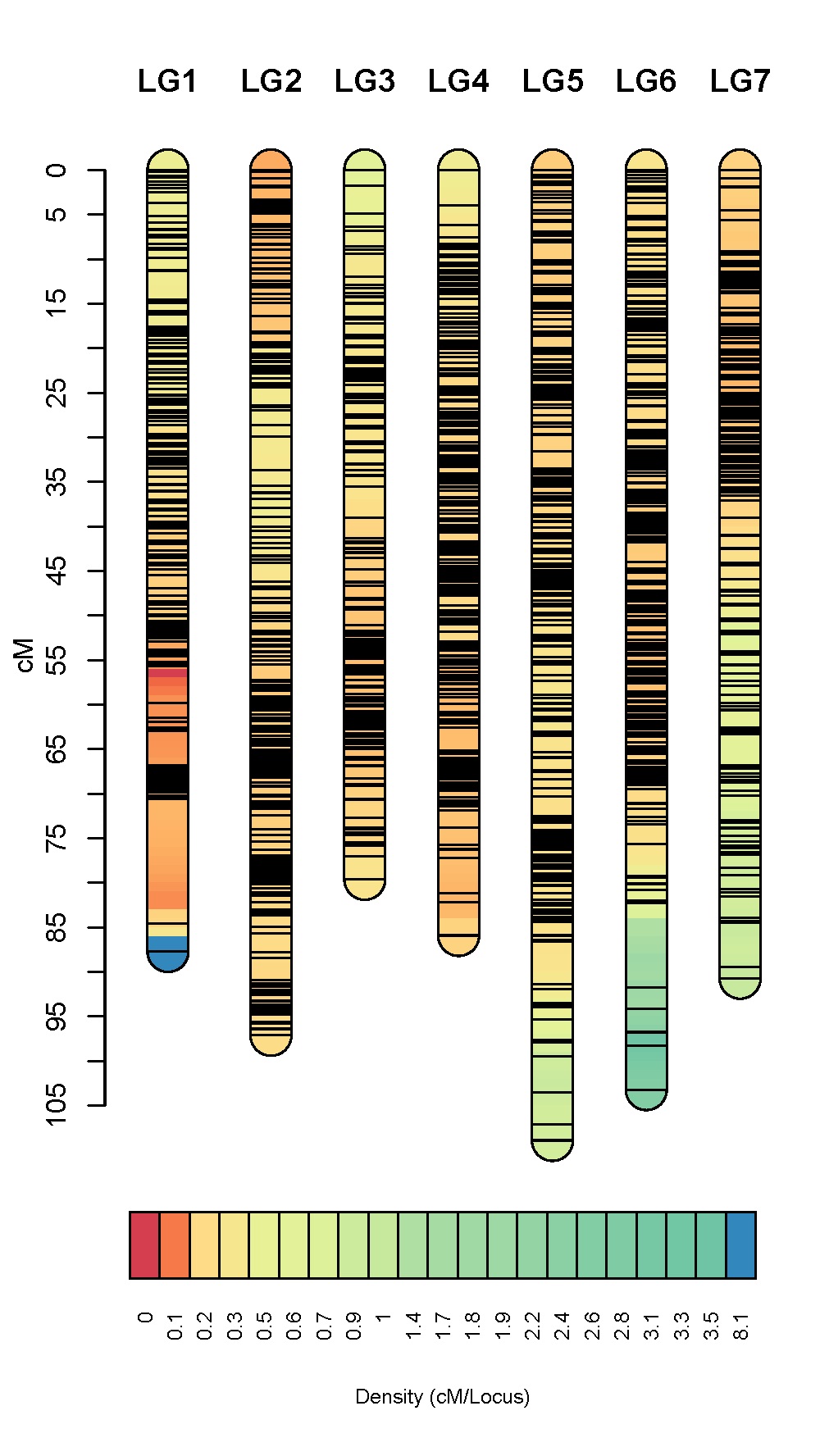

Supplement: Web_Material_uhac183 [file web_material_uhac183.zip › Fig. S7.jpg]

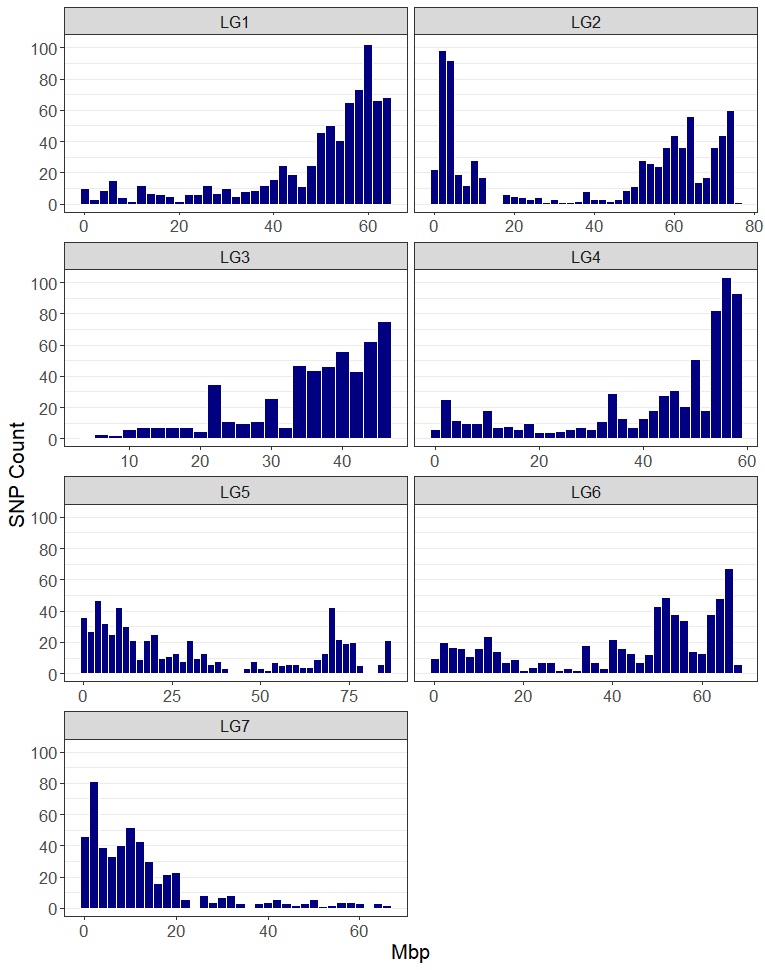

Supplement: Web_Material_uhac183 [file web_material_uhac183.zip › Fig. S8.jpg]

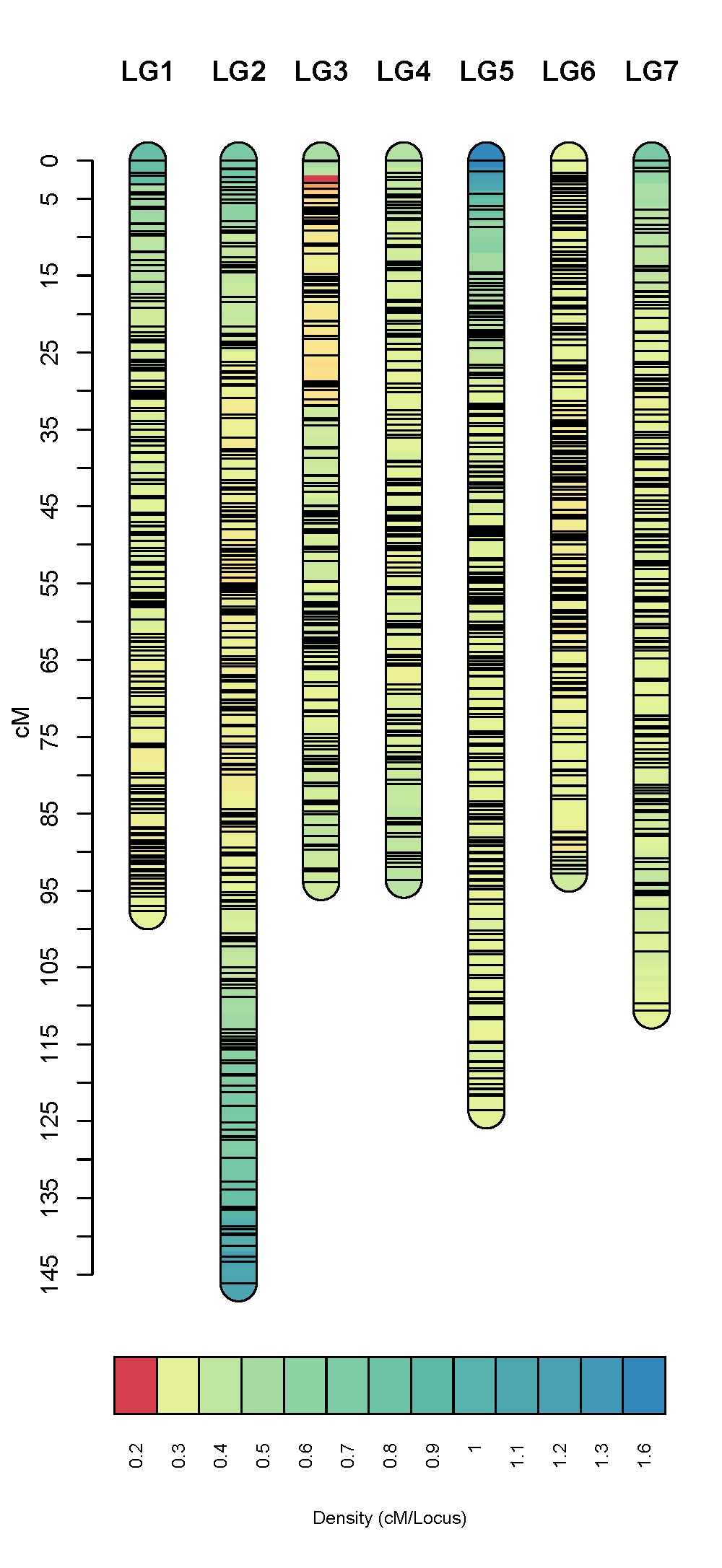

Supplement: Web_Material_uhac183 [file web_material_uhac183.zip › Fig. S9.jpg]
